# Longitudinal trajectories of left ventricular myocardial remodeling: associations with cardiovascular risk factors in the multi-ethnic study of atherosclerosis

**DOI:** 10.1016/j.jocmr.2025.101943

**Published:** 2025-08-22

**Authors:** Charlène A. Mauger, Bharath Ambale-Venkatesh, Avan Suinesiaputra, David A. Bluemke, Colin O. Wu, Joao A.C. Lima, Alistair A. Young

**Affiliations:** a School of Biomedical Engineering and Imaging Sciences, King’s College London, London, United Kingdom; b Department of Anatomy and Medical Imaging, University of Auckland, Auckland, New Zealand; c Department of Cardiology, Johns Hopkins Medical Centre, Baltimore, USA; d Department of Radiology, University of Wisconsin School of Medicine and Public Health, Madison, Wisconsin, USA; e Office of Biostatistics Research, National Heaart, Lung, and Blood Institute, National Institutes of Health, Bethesda, Maryland, USA.

**Keywords:** Shape Analysis, Cardiac remodeling

## Abstract

**Background:**

Understanding the influence of cardiovascular risk factors on longitudinal cardiac remodeling requires three-dimensional analysis of longitudinal shape changes beyond scalar indicators such as mass and volumes. The aim of this study is to determine trajectories of cardiovascular risk factor-related remodeling in a large cohort imaging study.

**Methods:**

We examined 2521 participants (54% female, aged 60 ± 9 years) of the multi-ethnic study of atherosclerosis (MESA) at baseline and after 10 years. Myocardial remodeling was assessed by longitudinal left ventricular shape trajectories derived from cardiac magnetic resonance imaging using a statistical shape atlas. Penalized logistic regression was used to examine the associations between trajectory scores and cardiovascular risk factors, after adjustment for sex and age at baseline. Multivariate regression was used to determine independent shape changes associated with each risk factor.

**Results:**

Between baseline and follow-up, there was a higher prevalence of hypertension (18.4%), antihypertensive medication usage (21.6%), statin usage, and treated diabetes mellitus (8.9%); all p<0.05. Longitudinal shape trajectory scores had stronger associations with obesity, high blood pressure, hypertension medication, and diabetes mellitus, than mass and volume changes (p<0.05). Multivariate regression showed independent longitudinal changes in wall thickening with obesity (13% increase), smoking (11% decrease), and high systolic blood pressure (5.6% increase), with distinct regional variations.

**Conclusion:**

Trajectories of cardiovascular risk factor-related longitudinal remodeling can be examined using shape atlases. In addition to global changes, each risk factor is associated with a distinct regional remodeling of the myocardium.

## Graphical abstract

**Figure:**
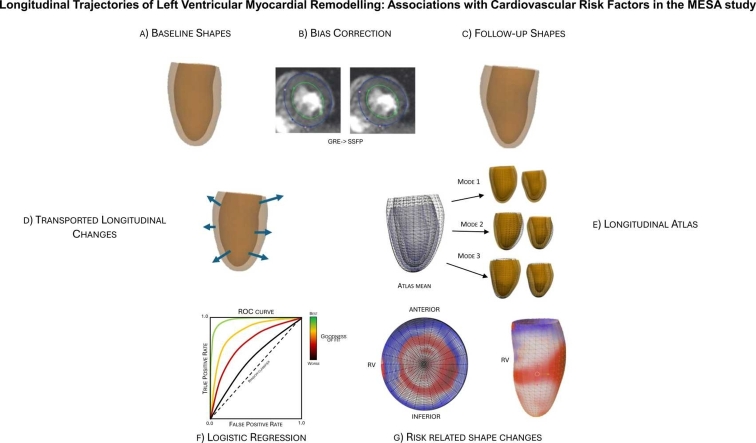

## 1 Background

The heart progressively undergoes alterations in structure and function over the life course in response to subclinical disease processes. Although cardiac remodeling has typically been characterized by changes in shape and function of the heart after cardiac injury [1], subclinical remodeling prior to the establishment of clinical symptoms in response to exposure to risk factors is indicative of pathophysiological processes leading to adverse outcomes and provides important information about heart health [2], [3]. Cardiovascular risk factors such as high cholesterol, hypertension, diabetes, obesity, and smoking are known to have adverse consequences on the aging heart by altering ventricular dimension, mass, and function [4], [5], [6]. As aging prolongs the exposure to cardiovascular risk factors, understanding the adverse effect on myocardial remodeling is important for the discovery of disease mechanisms.

Previous cross-sectional imaging studies have provided valuable information on ventricular mass and volume and their relationships with risk factors and incident events [7], [8], [9], [10], [11], [12], [13]. Longitudinal studies from the multi-ethnic study of atherosclerosis (MESA) have quantified changes in mass and volume associated with blood pressure and high-density lipoprotein (HDL) cholesterol concentration [14]. However, mass and volume measures do not capture the full range of heart shape adaptations arising due to aging and risk factor exposure. Furthermore, assessment of cardiac remodeling requires measurements and analysis of changes independent of inter-participant variations in geometry. Atlas-based analysis can provide detailed information on shape variations and their relationships with disease processes [3].

The aim of this study was to investigate three-dimensional (3D) longitudinal left ventricle (LV) changes associated with cardiovascular risk factors. We developed a novel analysis of subclinical remodeling that uses a statistical shape atlas of the longitudinal shape trajectories, corrected for baseline geometry. We characterized the LV longitudinal remodeling modes and used multivariate analysis to quantify the strength of associations between longitudinal trajectory scores and cardiovascular risk factors.

## 2 Methods

### 2.1 Study population

**Fig. 1:**
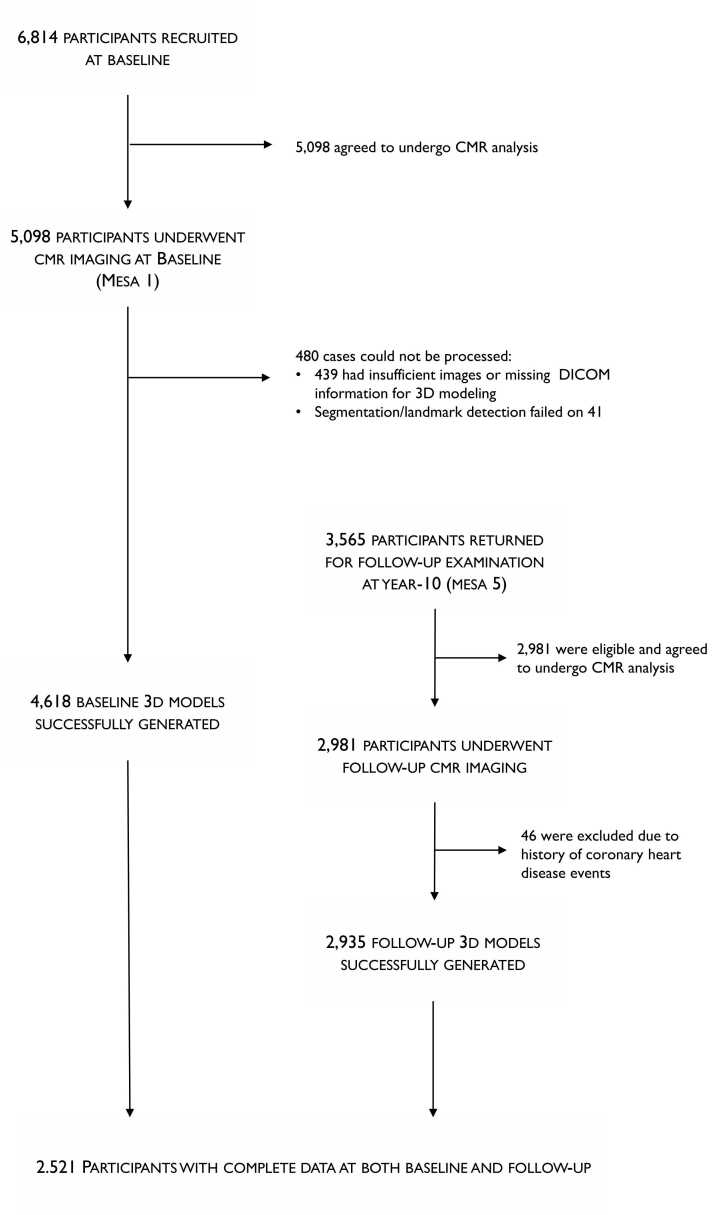
Flow chart diagram of study inclusion and exclusion criteria

Fig. 1 summarizes the data processing and participant data in this study, which were derived from the MESA study. The MESA study design and protocol have been described previously [15]. Briefly, 6814 men and women (45–84 years of age, 53% female) from six United States communities (Baltimore, Winston-Salem, Minneapolis, Los Angeles, Chicago, and New York) were enrolled between 2000 and 2002. All participants gave informed consent, and the study was approved by the institutional review boards of all MESA centers.

Participants were free of clinical cardiovascular disease at the baseline examination. Cine cardiovascular magnetic resonance (CMR) imaging was performed in 5098 participants at baseline and 3565 of these (70%) returned for a 10-year follow-up examination between April 2010 and February 2012. Of these, 2981 participants (84%) were eligible and consented to undergo CMR imaging, and 2521 had complete data at both baseline and follow-up.

### 2.2 CMR protocols

At baseline, CMR was performed on 1.5T Siemens and General Electric scanners. Details of the imaging protocol can be found in [16]. Images were acquired in four-chamber and two-chamber long axis, and short axis slices (spanning from LV apex to the mitral valve) with a gradient recalled echo (GRE) protocol (typical TR of 8–10 ms; TE of 3–5 ms, flip angle 20°), field of view 360–400 mm, pixel size from 1.4 to 2.5 mm, and slice thickness of 6 mm with 20–30 frames per slice (temporal resolution <50 ms).

Follow-up CMR imaging protocol was different from the baseline examination in that cine imaging was performed with a steady-state free precession (SSFP) protocol [18]. Images were acquired in four-chamber, two-chamber long axis, and short axis slices (spanning from LV apex to the mitral valve). All cine images had field of view 360 mm, 256 × 192 image matrix, slice thickness 8 mm with 2 mm gap, and an acquired temporal resolution of <40 ms, retrospectively reconstructed as 50 cine frames at 20–35 ms intervals over the cardiac cycle.

### 2.3 Measurement and definition of cardiovascular risk factors

Information on demographics, medical conditions, and family history was collected by questionnaire at the baseline examination. During baseline and follow-up examinations, height, weight, and waist and hip circumferences were measured. Blood was drawn for measuring lipids, inflammation, fasting glucose, fibrinogen, and creatinine. Resting blood pressure was measured three times in the seated position, and the average of the last two measurements was kept for the analysis. Glucose, triglycerides, HDL, and total cholesterol levels were also measured after 12 h of fasting. The Friedewald equation was used to calculate low-density lipoprotein (LDL) cholesterol concentrations. Participants were asked to report their sex as male or female. The cohort represents participants who self-identified as white, African American, Hispanic, or Asian, with most of the Asian individuals reporting Chinese ancestry. Details on data collected during the MESA examinations can be found in [15].

Seven categorical cardiovascular risk factors were selected for analysis in this study: obesity, high cholesterol, high blood pressure, smoking, diabetes, use of medication to treat hypertension, and use of medication to treat high cholesterol. All factors were categorized as binary variables. Obesity was defined as body mass index (BMI) more than 30 kg/m^2^. Hypertension was defined as hypertension diagnosis or systolic blood pressure (SBP) >140 mmHg and diastolic blood pressure (DBP) >90 mmHg at examination. Diabetes was defined as physician diagnosis of diabetes or impaired fasting glucose (from 100 to 125 mg/dl). High cholesterol was defined as total cholesterol >240 mg/dl, LDL cholesterol >160 mg/dl, HDL cholesterol <40 mg/dl, and triglycerides >150 mg/dl. Participants smoking at the time of follow-up were defined as smokers.

### 2.4 Shape trajectory analysis

**Fig. 2:**
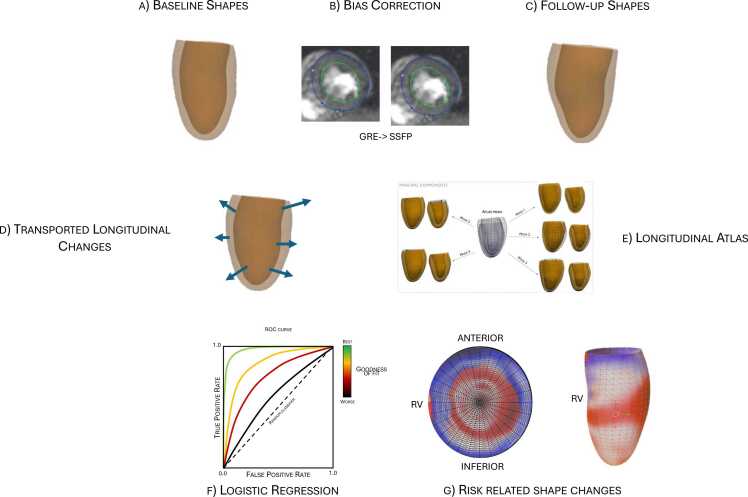
Overall pipeline. *GRE* gradient recalled echo, *SSFP* steady-state free precession

The overall processing pipeline from the LV shape model to computation of risk-related shape changes is shown in Fig. 2. First, an LV shape model was customized to CMR images for both baseline and follow-up exams (Fig. 2a). Due to different imaging protocols, baseline shape models (derived from GRE CMR images) were transformed to the equivalent SSFP-derived shape models to correct for the systematic differences (Fig. 2b). The differences between baseline and follow-up shapes were computed using the parallel transport method (Fig. 2d). A longitudinal shape change atlas was constructed using principal component analysis (PCA), applied to the 3D personalized transported shape trajectories (i.e. shape changes added to common reference baseline shape, Fig. 2e). Details of each step are given below.

#### 2.4.1 Baseline LV shape models

The automated processing pipeline described in [17] was used to reconstruct LV shapes from baseline CMR images. Shape models were evenly sampled at sufficient resolution to capture all the shape information, resulting in 1562 3D Cartesian points. Only end-diastole (ED) and end-systole (ES) models were included.

#### 2.4.2 Follow-up LV shape models

Manual analyses of the follow-up CMR examinations [18] were performed as described previously in [19] using Cardiac Image Modelling software (version 6.2; Auckland MR Imaging Research Group, University of Auckland, Auckland, New Zealand). To avoid bias due to the analysis process and correct for breath-hold misregistrations, contours for the follow-up cases were passed through the same automated model shape analysis pipeline as used for the baseline cases.

#### 2.4.3 Bias correction

The change from baseline GRE to follow-up SSFP imaging introduced measurement bias due to differences in image appearance, requiring a post-hoc correction for LV remodeling analysis. SSFP is known to provide larger LV cavity volume and smaller LV mass than GRE [20]. This is also manifest as a regional shape bias in the personalized shape models and must be corrected locally at each point in the shape model [21]. We adapted the method of [22] to correct for this shape bias using nearly 500 randomly selected participants who were scanned during the 10-year follow-up exam with both GRE and SSFP protocols.

LV personalized shape models were generated for those participants for both acquisitions. For the GRE acquisitions, the same processing pipeline used for the baseline exam was performed (deep learning generation of 3D contours and landmarks followed by shape model customization). For the SSFP acquisitions, the follow-up exam analysis and contour fitting protocol described above was used to customize the shape models.

GRE personalized shape models were aligned to a mean ED shape using generalized Procrustes analysis. To retain contraction-related transformations, ED and ES models were concatenated into a single shape per participant. To reduce the dimension of the problem, PCA was applied to the aligned GRE models, and SSFP personalized shape models were projected onto this PCA atlas to obtain GRE-derived scores. Partial least-squares regression (PLSR) was then used to estimate the SSFP projected scores from the GRE PCA scores. ED and ES timepoints were manually selected by expert readers for both GRE and SSFP acquisitions, ensuring consistency across imaging protocols. This expert-driven selection helps mitigate variability introduced by differences in temporal resolution and reconstruction, and is inherently accounted for in the shape correction model. Fig. S1 summarizes the bias correction pipeline.

Leave-one-out cross-validation was used to evaluate bias correction performance by training on all but one case and computing errors in LV volumes, mass, and surface position and resemblance on the held-out case. This was repeated for all cases, and errors were averaged. Results of this method are provided in the Supplementary Materials.

#### 2.4.4 Atlas trajectory scores

A crucial step in studying longitudinal changes is the management of cross-sectional variation. As the cross-sectional variation (across participants) is generally larger than the variation within participants (across longitudinal exams), it is important to remove any cross-sectional variation so that longitudinal changes can be examined. To remove the contribution of baseline shape, we mapped the remodeling trajectories for each participant onto a common reference, i.e., the mean baseline shape. The mapping was performed using parallel transport, which is a common method for studying shape changes [23], [24], [25]. This enables the building of subject-specific remodeling trajectories starting from the same common reference (mean baseline shape), which eliminates cross-sectional variation, allowing analysis of longitudinal variation.

A remodeling trajectory is first represented as the difference between baseline and follow-up shapes. We calculated each participant’s change in shape between baseline and follow-up by subtracting the baseline shape from the follow-up shape, resulting in a set of 3D displacement vectors (one for each 3D point in the shape model). The set of displacement vectors for each participant is the remodeling trajectory. Using linear shift, the trajectory was then transported to the common reference, defined as the mean baseline LV shape. Linear shift is a form of parallel transport appropriate for analyzing shape changes which are not far from each other (i.e., can be considered as belonging to the same linear tangent space in the shape manifold [22], [23], [24]). PCA scores (as z-scores) were computed from the aligned trajectories across all participants. These were then used as remodeling trajectory scores. Details of this procedure are provided in the Supplementary Material.

### 2.5 Association of trajectories with cardiovascular risk factors

The strength of the relationship between the LV shape changes and risk factors was evaluated using penalized logistic regression (LR). Two LR models were compared for each risk factor using the presence of the risk factor as the categorical response variable. A participant was categorized as positive if the risk factor was present at follow-up and negative if the risk factor was not present at both baseline and follow-up. The first model, called the Mass-Volume model, used differences in LV mass, volumes, and ejection fraction (EF) between follow-up and baseline as predictor variables. The second model, called the Atlas model, used trajectory scores of the longitudinal shape change atlas as predictor variables. The number of principal components included in the Atlas model accounted for 99% of the total variation. Both Mass-Volume and Atlas models were adjusted for sex and age at baseline. Additionally, models adjusted for age only were used to evaluate the strength of association with sex, considered as a categorical response variable.

The LR models were trained using ten-fold cross validation to avoid comparison bias due to training/test data selection. The same seeds were given to the random generator for each fold to make sure that the train-test splits were always deterministic and consistent between the two LR models. For each fold, the dataset was split in a stratified fashion into 80% training and 20% testing. This stratified 10-fold cross validation was defined to ensure that the training and test sets have the same proportion of risk-positive cases as in the original dataset for each fold, and to ensure that the validation result was a close approximation of generalization results. Note that there was an imbalanced proportion between risk and negative cases in the dataset.

**Fig. 3:**
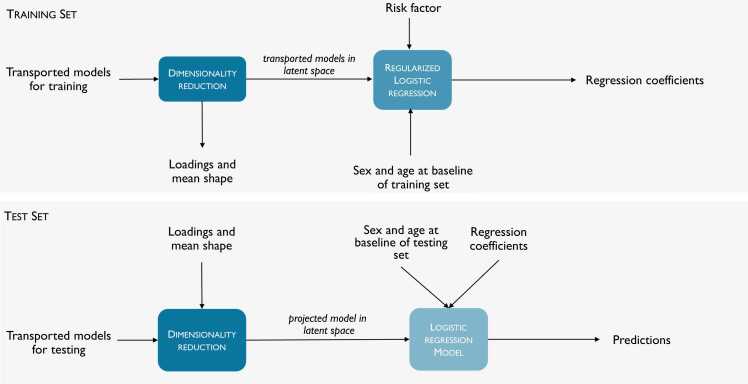
Optimization framework

Training sets were used for model construction of the LR models. Hyperparameters of the logistic regression models included elastic-net mixing parameter, the contribution of the L1 and L2 penalties, and the regularization strength, which were all optimized using randomized grid search with a five-fold stratified cross validation. Balanced accuracy score was used as the performance metric. Balanced accuracy is defined as the average recall obtained on each class and can therefore deal with imbalanced datasets. The best value is 1 (perfect discrimination) and the worst value is 0 (no discrimination). Test datasets were used to evaluate the model prediction performance. Cross-validations, PCA, and LR hyperparameters tuning were implemented in Python using the scikit-learn package. This pipeline is shown in Fig. 3.

### 2.6 Risk-related remodeling modes

Association between LV longitudinal changes and cardiovascular risk factors was further examined using multiple linear regression for each risk factor (Fig. 2g). Response variables were the atlas trajectory scores, while predictor variables were age at baseline, variation in BSA, and presence of the cardiovascular risk factors. Longitudinal effects of SBP, DBP, BMI, HDL, LDL, race/ethnicity, smoking status, sex, diabetes, usage of statins, and usage of hypertensive medication were analyzed. Race/ethnicity was included as a set of dummy variables, with individuals who self-identified as white were used as the reference category. Race/ethnicity was mutually exclusive.

For each risk factor, a risk-related remodeling mode was estimated by partitioning the contribution of each factor to the shape. Confounding factors such as age at baseline and multiple risk factors were accounted for as covariates. For each risk-related remodeling mode, risk-related changes in conventional measures such as end-diastolic volume (EDV), end-systolic volume (ESV), ejection fraction (EF), LV mass (LVM), longitudinal shortening (LS), LV sphericity, relative wall thickness (RWT), LV anteroseptal wall thickness, inferolateral wall thickness, and LV mass-to-volume ratio were also computed for the purposes of interpretation.

### 2.7 Statistical analysis

Continuous variables, expressed as mean ± standard deviation, were compared using a Student’s t-test or Welch’s t-test depending on the population’s variance. Categorical variables were expressed as frequency and compared using a Pearson's chi-squared test. A p-value <0.05, after Bonferroni correction, was deemed significant for this study.

## 3 Results

### 3.1 Study population

Fig. 1 shows a flow chart illustrating data inclusion and exclusion criteria used in this study. Of the 2981 participants with both baseline and follow-up CMR examinations, 46 were excluded due to incident coronary heart disease or heart failure events. Of the remaining 2935 participants, while all had CMR data at both time points, 3D shape models were successfully generated at both baseline and follow-up for 2521 participants. The remaining 414 participants were excluded from the 3D shape analysis due to insufficient image data or failure of landmark detection or segmentation at baseline.

Table 1 shows differences in population characteristics between baseline and follow-up groups. LV volumes were extracted from the personalized shape models. The average age of participants in the MESA baseline examination was 59.6 ± 9.4 years, and 54.3% were female. There was a significant increase in the prevalence of hypertension (18.4%), antihypertensive medication usage (21.6%), statins usage (21.7%), and treated diabetes mellitus (8.9%); all p<0.05.

**Table 1:** Population characteristics.

N = 2521	Baseline (Year 0)	Follow-up (Year 10)
Age (y)	59.6 ± 9.4	69.1 ± 9.2*
Women/Men	1370 (54.3)/1151 (45.7)	1370 (54.3)/1151 (45.7)
Race/ethnicity (%)		
White	1047 (41.5)	1047 (41.5)
Asian	337 (13.4)	337 (13.4)
African American	618 (24.5)	618 (24.5)
Hispanic	519 (20.6)	519 (20.6)
Current smoker	259 (10.5)	174 (7.1)*
Body mass index (kg/m^2^)	27.7 ± 4.8	27.8 ± 5.1*
Systolic blood pressure (mmHg)	123.3 ± 20	123.3 ± 20.3
Diastolic blood pressure (mmHg)	71.8 ± 10.1	68.3 ± 9.9*
Total cholesterol (mg/dl)	194.8 ± 35	184.4 ± 36.8*
LDL (mg/dl)	117.8 ± 30.9	106.8 ± 32.2*
HDL (mg/dl)	51.6 ± 15.4	56.1 ± 16.7*
Taking medication		
Hypertension	768 (31.2)	1301 (52.8)*
Lipidaemia	331 (13.4)	865 (35.1)*
Diabetes status:		
Impaired fasting glucose	286 (11.6)	519 (21.1)*
Untreated diabetes	33 (1.3)	44 (1.8)*
Treated diabetes	154 (6.2)	372 (15.1)*
Diagnosed hypertension	931 (37.8)	1407 (57.1)*
Left ventricular function		
End-diastolic volume (mL)	129 ± 28	118 ± 31*
Mass (g)	119 ± 27	122 ± 33*
End-systolic volume (mL)	48 ± 13	46 ± 18*
Ejection fraction (%)	63 ± 6	62 ± 7*
Mass-to-volume ratio (g/mL)	1.0 ± 0.2	1.1 ± 0.2*

Values are mean ± standard deviation for numerical variables or n (%) for categorical variables. *LDL* low-density lipoprotein, *HDL* high-density lipoprotein

* p<0.05 for the paired t test to evaluate differences from baseline to follow-up or chi-square tests to evaluate the differences for categorical variables

On average over 10 years, there was a 4% reduction in LV EDV (p<0.001), while LV mass increased by 1% (p<0.001) and the LV mass to volume ratio increased by 10% (from 1.0 to 1.1) (p<0.001). Additionally, there was a slight but statistically significant decrease in EF by 1.7% (p<0.001).

### 3.2 Atlas trajectory scores

**Fig. 4:**
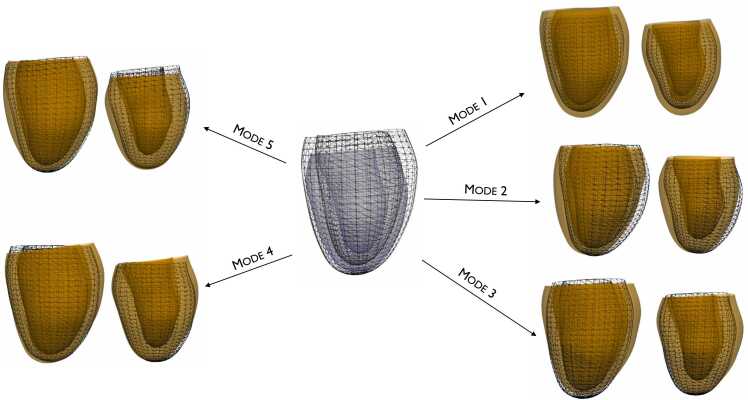
First five principal components of left ventricular longitudinal remodeling. The mean shape is shown in the center (ED: wireframe, ES: gray surface). For each mode, ED is shown on the left and ES is on the right. −2 standard deviation from the average shape is shown in wireframe and +2 standard deviation is shown with a surface. *ED* end-diastole, *ES* end-systole

Fig. 4 visualizes the first five principal components of the longitudinal shape change atlas by showing the shapes at ±2 standard deviations from the average shape. These first five modes represent the biggest longitudinal shape change variations of the LV across the 2521 cases, accounting for 61.6% of the total variation. Additionally, since ED and ES shapes were concatenated in the PCA calculation, Fig. 4 also displays variations in motion between ED and ES frames. The biggest variation was associated with the changes in dimension and sphericity of the LV over time, representing 21.9% of the total variation. The second mode was mostly correlated with changes in septal wall thickness with constant volume over time and accounted for 9.7%. The third principal component (8.8% of the total variation) was positively correlated with an increase in LV cavity size, with constant mass, and negatively correlated with changes in mass-to-volume ratio and relative wall thickness (concentric remodeling). Accounting for 7.9%, the fourth component was associated with a decrease in wall thickness and ejection fraction (eccentric remodeling), while the fifth principal component (accounting for 6.1%) was mostly associated with function changes (change in EF, wall thickening, and shortening).

### 3.3 Relationship with cardiovascular risk factors

Table 2 shows the results of the cross-validation LR models on the testing datasets, comparing predictors comprising longitudinal shape change atlas scores extracted from the transported shape changes (Atlas) with a baseline model (Mass-Volume) using predictors comprising changes in mass, volumes, and ejection fraction between baseline and follow-up, with both models including sex and age at baseline as predictors.

**Table 2:** Balanced accuracy comparison between 2D measurements and shape measurements, adjusted for sex and age at baseline.

	Mass-Volume	Atlas
Diagnosed hypertension	0.52 ± 0.03	0.65 ± 0.03*
Obesity	0.58 ± 0.03	0.71 ± 0.01*
Hypertension medication	0.54 ± 0.05	0.69 ± 0.04*
Use of statins	0.51 ± 0.03	0.51 ± 0.02
Smoking	0.57 ± 0.03	0.62 ± 0.03
Sex	0.66 ± 0.02	0.80 ± 0.01*
High cholesterol	0.64 ± 0.03	0.65 ± 0.03
Diabetes	0.61 ± 0.04	0.68 ± 0.05*

* indicates p<0.05. Mass-Volume model predictor variables were ΔEDV + ΔESV + ΔM + ΔEF + sex + age at baseline. Atlas model predictor variables were atlas trajectory scores + sex + age at baseline. Balanced accuracies are written as mean ± standard deviation over the 10 folds.

The Atlas model showed stronger associations compared with the Mass-Volume model for obesity, diabetes, diagnosed hypertension, and hypertension medication. Also, the Atlas model showed significantly higher balanced accuracy for sex compared with the Mass-Volume model, when using sex as a response variable.

### 3.4 Age and sex related remodeling modes

**Fig. 5:**
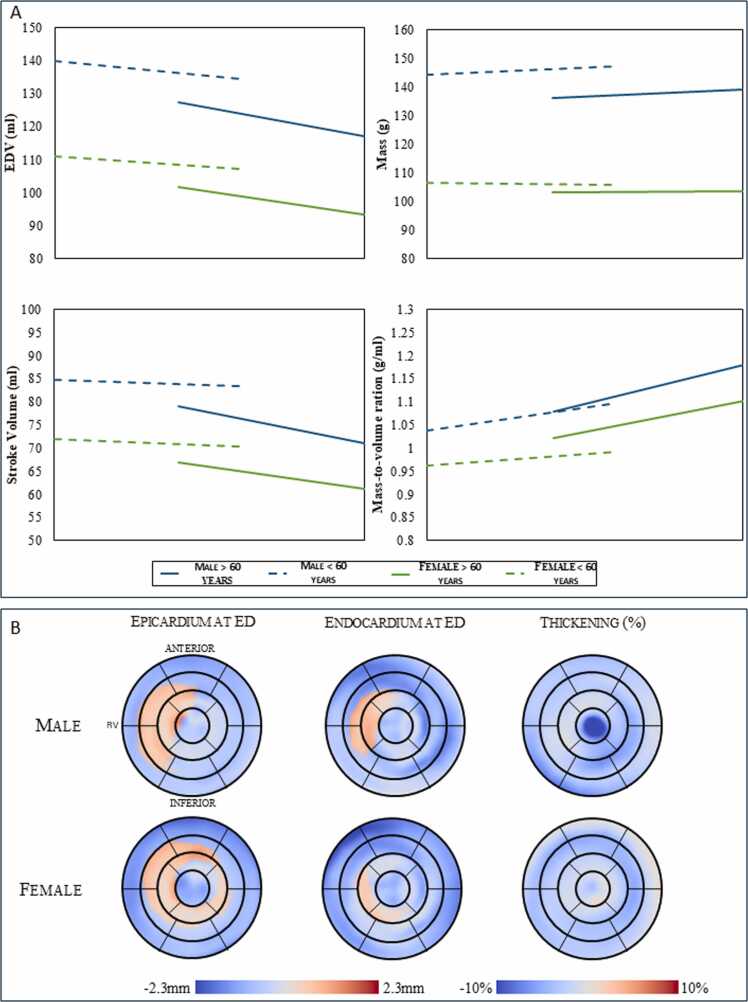
Age-related cross sectional and longitudinal changes of left ventricular geometry by sex. (A) longitudinal changes expressed as mass, volumes, and sphericity: each line segment represents atlas-derived longitudinal changes for the two age groups and two sex groups. The trend between two successive lines represents cross-sectional variations amongst the age groups calculated from the 3D CMR-derived LV models at baseline. (B) Regional longitudinal changes between age groups. *ESV* end-systolic volume, *EDV* end-diastolic volume. Regional wall thickening percentage was calculated as 100 x (Wall thickness at ES - Wall thickness at ED) / Wall thickness at ED

Fig. 5A shows age-related and sex-specific LV remodeling modes between baseline and follow-up, derived from our regression models and remodeling modes. Each line segment represents atlas-derived longitudinal changes for the two age groups (<60 years and >60 years) and the two sex groups. Table 3 shows those longitudinal variations expressed as percentages for the two age groups and different sexes. Only values above the standard error of mean change are shown. In the table, a positive value represents an increase over time, while a negative value indicates a decrease overtime.

**Table 3:** Age-related longitudinal variations (%) in LV geometry stratified by sex.

Sex	Age group	EDV	ESV	Mass	EF	MVR	SphVi		LS	RWT
							ED	ES		
Male	< 59.6 y	−4.0	−7.8	2.0	1.7	5.5	−3.1	−7.2	−0.7	13.4
	> 59.6 y	−8.6	−4.5	2.2	−2.0	8.6	−4.6	−4.5	−7.6	13.3
Female	< 59.6 y	−3.9	−6.5	−0.5	1.3	3.0	0.3	−2.8	−1.4	7.1
	> 59.6 y	−9.0	−7.8	-	−0.6	7.3	−0.5	−2.6	−6.9	7.2
SEM		0.4	0.6	0.3	0.2	0.4	0.3	0.5	0.5	0.1

*EDV* end diastolic volume*, ESV* end-systolic volume*, EF* ejection fraction*, MVR* mass-to-volume ratio*, SphVi* sphericity volume index*, LS* longitudinal shortening*, RWT* relative wall thicknes*s, ED* end-diastole*, ES* end systole*.* Only values above the standard error of mean change (SEM) are shown. A positive value indicates an increase overtime, while a negative value indicates a decrease overtime

There was a longitudinal increase in mass for men (2% over 10 years), with older men having a bigger increase (from 2.0 to 2.2%). This stands in contrast to a cross-sectional decrease with older men having lower LV mass compared to younger men (136 g vs 144 g, respectively p<0.001). There is a cohort effect underlying LV mass in men, which operates in the opposite direction to the age effect [18]. As for women, there was a slight longitudinal decrease in LV mass for the younger group and a slight aging decrease when viewed cross-sectionally (106.3 g vs 103.2 g, p = 0.01). LV EDV and LV ESV decreased over 10 years in both men and women, older women and men having smaller cavity volumes compared to their younger counterparts (93.2 ml vs 107 ml (p<0.001) and 117 ml vs 134 ml (p<0.001) at ED respectively and 34.6 ml vs 39.1 ml (p<0.01) and 48.3 ml vs 55.1 ml (p<0.001) at ED respectively). EDV reduction was greater in the older group.

Fig. 5B shows regional sex-specific LV remodeling trajectories between baseline and follow-up, derived from our regression models and remodeling modes on the 17 American Heart Association segments. Among females, aging was correlated with a decrease in wall thickening, primarily in the midwall of the LV. This reduction was found to be less pronounced compared to that observed in males. Males exhibited a decrease in wall thickening, predominantly localized in the apical and mid-lateral regions.

### 3.5 Race/ethnic related LV remodeling modes

Table 4 summarizes ethnic/race-related LV remodeling modes between baseline and follow-up, derived from our regression models and remodeling modes. Reference values are for White Americans. Asian Americans demonstrated bigger increase in EDV (+3.4%) and a bigger decrease in MVR (−3%) and RWT (−3.4%) over time. In contrast, African Americans showed modest increases in EDV (+0.6%), along with increases in both MVR (+2.8%) and RWT (+3.3%), indicating a shift toward concentric remodeling. Hispanics exhibited a substantial decrease in EDV (−3.6%) and concurrent increases in MVR (+4.2%) and RWT (+3.1%), reflecting a pattern of concentric remodeling with reduced ventricular filling.

**Table 4:** Ethnic/race-related longitudinal variations (%) in LV geometry.

Ethnicity/race	EDV	ESV	Mass	EF	MVR	SphVi		LS	RWT
						ED	ES		
Asian American	3.4	-	0.4	2.2	−3.0	-	−5.5	−3.5	−3.4
African American	0.6	2.5	3.4	−1.1	2.8	−2.2	-	-	3.3
Hispanic	−3.6	−1.6	0.4	−1.2	4.2	−2.2	−1.4	−2.9	3.1
	0.4	0.6	0.3	0.2	0.4	0.3	0.5	0.5	0.1

*EDV* end diastolic volume, *ESV* end-systolic volume, *EF* ejection fraction, *MVR* mass-to-volume ration, *SphVi* sphericity volume index, *LS* longitudinal shortening, *RWT* relative wall thickness, *ED* end-diastole, *ES* end systole. Only values above the standard error of mean change (SEM) are shown. A positive value indicates a bigger increase overtime compared to the reference ethnicity/race (white American) used in the regression model, while a negative value indicates a smaller decrease overtime compared to the reference ethnicity/race

### 3.6 Risk-related LV remodeling modes

Table 5 summarizes the associations between the risk factors and atlas-derived longitudinal variations in LV geometry. Supplemental Fig. S4 shows the remodeling mode for each risk factor. Each risk model was adjusted for age, gender, race/ethnicity, body-surface-area variation, and all other risk factors in the table.

**Table 5:** Changes in longitudinal LV geometry in relation to cardiovascular risk factors from our multivariate analysis.

	Longitudinal variations over 10 years (%)								
	EDV	ESV	Mass	EF	MVR	SphVi	SphVi	LS	RWT
						ED	ES		
SBP	4.7		6.7	2.4	2.0	0.8	−3.7	−0.6	5.6
DBP	−6.8		−1.5	−3.7	5.7	−4.2	1.1	−2.9	−0.6
Medications									
Hypertension		−4.6	−2.4	2.4	−1.7	1.5	−2.7		2.9
Hyperlipidaemia	−0.5	0.1		−0.3	0.7	−1.1		1.5	−0.9
Smoking status	−4.8		0.9	−2.4	5.5	−2.0	1.1	−2.8	−1.4
BMI	−1.2	−3.6	1.0	1.5	1.7	−0.3	−2.5	1.2	3.0
Diabetes mellitus	−2.9	−1.1	0.8	−1.0	3.8	−1.8	−1.1	−2.6	1.6
LDL	−1.1		0.9	−0.4	2.0	−1.7	−1.6	−1.3	0.8
HDL	0.7			0.7	−1.1	0.5		0.6	0.9

*EDV* end diastolic volume, *ESV* end-systolic volume, *EF* ejection fraction, *MVR* mass-to-volume ration, *SphVi* sphericity volume index, *LS* longitudinal shortening, *SWT* septal wall thickness, *IWT* inferolateral wall thickness, *RWT* relative wall thickness, *ED* end-diastole, *ES* end systole. Only values above the standard error of mean change are shown. Negative values indicate a decrease overtime

Longitudinal changes with SBP were positively associated with changes in LV mass, volumes, MVR, RWT, and EF. LV volumes were smaller in diabetic participants, but LV mass and MVR were larger than those without diabetes. Decreases in LV volumes were observed in individuals with lower diastolic blood pressure, BMI over 30, and smokers. Smoking status and diastolic blood pressure showed the greatest increase in MVR. BMI and hypertensive medication were associated with increased and decreased LV mass, respectively. An increase in EF was associated with higher SBP and hypertension medication use, while a decrease in EF overtime was linked to smoking status and higher DBP. DBP and smoking status were associated with decreases in sphericity at ED and increases in sphericity at ES. Diabetes and LDL were associated with decreases in both ED and ES sphericity.

Regarding regional changes, SBP, hypertensive medication, and HDL were associated with a global increase in wall thickening. HDL was associated with a greater increase in thickening in the mid/basal lateral regions. Hypertensive medication had greater increase in the mid-wall lateral region and the apical region. SBP showed the greatest decrease of all three, with localized reduction also observed in the lateral regions.

LDL was associated with a global decrease in wall thickening, except near the apical region where an increase in thickening is shown. The greatest decrease is shown in the mid/basal inferolateral regions. Smokers showed a reduction in the lateral and apex regions, while DBP was associated with a bigger decrease in the anterior regions.

Both decreases and increases in myocardial wall thickening were observed in diabetics participants and participants taking statins. In diabetics, the greatest decrease happened in the basal infero- and anteroseptal regions. The midwall showed a slight increase in thickening. Participants taking statins showed an increase in the basal regions and apical regions and a decrease in the mid regions.

Obese participants and smokers showed the biggest changes in wall thickening, accounting for 13% and −11%, respectively.

## 4 Discussion

Cardiovascular risk factors such as diabetes mellitus, obesity, smoking, hypertension, and hyperlipidaemia are known to affect ventricular mass and volume before the manifestation of clinical symptoms. These changes are often referred to as myocardial remodeling [4], [5]. However, mass and volume remodeling metrics do not capture all the available geometric or functional information present in modern medical imaging examinations. Furthermore, confounding effects of age, sex, and other risk factors have hindered understanding of the remodeling processes undergone. We assessed the subclinical correlates of longitudinal LV shape changes over a 10-year period in 2521 participants of the Multi-Ethnic Study of Atherosclerosis using atlas-based analysis. We found that longitudinal shape trajectory scores derived from the 3D atlas had stronger associations with risk factors of obesity, high blood pressure, hypertension medication, and diabetes mellitus than mass and volume changes. Regional and global shape changes computed from multivariate linear regression, corrected for confounders and other risk factors, enabled estimation of global and regional variations in remodeling with healthy age and sex (Table 5, Fig. 5), as well as remodeling with risk factors (Table 5 and Supplementary Figures). These show for the first time how remodeling processes act differently in different regions.

### 4.1 Age and sex related LV remodeling

Better comprehension of changes in cardiac structure and function associated with sex and aging could enable reduction in disparity of outcomes, particularly between sexes. In our study, differences in LV remodeling trajectories between sexes and older and younger participants were observed. Fig. 5 shows inward remodeling at ED except in septal mid and apical regions, and outward remodeling at ES except at apical septal and inferior base regions. This is consistent with a significant age-related decrease in LV cavity dimension observed in both sexes across the two age groups. Those changes lead to an increasing mass-to-volume ratio over time, consistent with concentric remodeling as found in previous studies of mass and volume changes in MESA [14], [18], [26] and resemble what might be expected for the evolution of HF with preserved EF. This is in accordance with recent research indicating that LV hypertrophy is more prevalent in older individuals [18] as it is an adaptive response to an increased cardiac afterload caused by a loss of vascular compliance and an age-related arterial dilatation.

We also found that cardiac contraction, here demonstrated by EF and LS, was better preserved in women, consistent with findings from [27] suggesting that the female hearts have 10–24% larger contractility than male hearts. Men conversely showed a mean decrease in both ED and ES sphericity, known to be a predictor of heart failure, cardiovascular disease and coronary heart disease [28].

### 4.2 Ethnic differences in LV remodeling

Our findings reveal substantial ethnic disparities in longitudinal left ventricular remodeling patterns that may contribute to known differences in cardiovascular outcomes. African Americans demonstrated the most pronounced changes in LV geometry, with significant increases in both LV mass and relative wall thickness over the 10-year follow-up period, consistent with concentric remodeling patterns previously associated with increased risk for heart failure with preserved ejection fraction [41], [42].

Hispanic participants also showed trends toward left ventricular hypertrophy and abnormal remodeling, consistent with (Rodriguez et al., 2010, [43]). In contrast, Chinese Americans consistently maintained lower RWT changes throughout the observation period, potentially reflecting cardioprotective genetic or lifestyle factors (Post et al., 2022). These distinct ethnic remodeling signatures align with previous analyses from cross-sectional results on the MESA cohort but also provide novel insights into the temporal progression of these differences. Understanding these ethnicity-specific remodeling patterns is essential for developing tailored approaches to cardiovascular risk assessment and management across diverse populations.

### 4.3 Cardiovascular risk factor-related LV remodeling

Cross-sectional studies have shown that LV geometry is highly influenced by blood pressure, obesity, and other risk factors [29], [30], [31]. Here, we showed that these factors play a crucial role in the longitudinal trajectory of LV geometry and function over time, when corrected for the confounding effects of covariates and other risk factors.

Diabetes has been shown to correlate with an increased incident of congestive heart failure. In our study, participants with diabetes were found to have a greater diminution of the LV chamber and sphericity at both ED and ES compared to non-diabetic participants. Supplemental Fig. S4 also shows inward remodeling in basal regions and outward at the apex at ED, consistent with reduced sphericity. In addition, there was a slight increase in mass and a steeper increase in RWT, indicating a shift towards concentric remodeling. This factor could be one of the reasons behind the heightened susceptibility of diabetic individuals to overt coronary heart disease and heart failure. These results are supported by other established population-based studies. In the Framingham study, a decrease in LV volumes of diabetics has been shown to happen at a steeper rate than individuals without diabetes, while LV mass increases at a steeper rate [32], [33], [34], [35], [36].

Diabetics also had lower longitudinal shortening, decreasing at the steepest rate. Interestingly, ejection fraction was not impacted. Other studies have also reported impaired longitudinal shortening in diabetics [35], [37]. Diabetic participants also displayed a faster decrease in LV sphericity similar to [38] in the MESA cohort. The mechanisms of impaired glucose metabolism on the LV geometry are yet to be identified. Heerebeek *et al.* [38] suggested that increased LV stiffness, induced by an accumulation of collagen and advanced glycation end products, contributes to LV remodeling. Our results indicate that these effects may be regional rather than global.

We observed (Supplemental Fig. S4) that obesity, as indicated by BMI, was associated with an inward endocardial remodeling at ED and ES in the apical and midwall region, with mixed inward and outward epicardial remodeling consistent with a longitudinal increase in LV mass and wall thickness and a decrease in LV volume.

This finding is consistent with previous reports of obesity as an independent risk factor for LV hypertrophy.

In contrast, in the Framingham study, increase in BMI over time was related to increased LV mass and volume [33]. The discrepancies between studies may be related to a sex-specific remodeling pattern. Rider *et al.*’s [39] findings suggest that obese males tend to display mainly concentric hypertrophy, whereas obese females exhibit both eccentric (increase LV volume mass) and concentric hypertrophy (increase in mass with smaller cavity size). However, unlike existing remodeling classifications, we demonstrated here that there is a strong and distinct regionality to the homeostatic response of the myocardium in which concentric and eccentric adaptations occur concurrently. Increased BMI is associated with concentric hypertrophy of the mid inferior wall and eccentric hypertrophy of the anterior and lateral basal walls.

Elevated blood pressure is widely recognized as the primary predictor of LV hypertrophy and generally leading to concentric remodeling patterns. For SBP, Supplemental Fig. S4 shows outward remodeling at epicardium and endocardium at ED, with increased wall thickening, consistent with an increase in LV mass and EDV over time.

Conversely, DBP was associated with an inward remodeling at ED on both epicardial and endocardial surfaces, consistent with reduction in EDV. This is consistent with previous findings [2], [4], [6]. In the CARDIA study [6], SBP correlated with increased LV mass and EDV while DBP was inversely correlated with LV EDV volume. SBP was more strongly correlated than DBP to the LV mass. This is consistent with findings from the CARDIA study [6] and other MESA studies [18].

We found a positive association between smoking and LV mass and thickness. Smoking was associated with longitudinal decrease in LV volume.

One possible explanation is that smoking may be associated with myocardial fibrosis, which affects LV cavity volume [40]. Smoking was associated with increasing LV mass in the longitudinal Framingham study, findings in accordance with several cross-sectional studies [33]. Supplemental Fig. S4 shows that, concentric hypertrophy was identified in the mid-anterior wall only.

### 4.4 Clinical relevance

Identifying risk factor-specific remodeling patterns can provide valuable insights for tailoring personalized prevention and treatment strategies. Understanding how different cardiovascular risk factors influence longitudinal LV remodeling, may lead to better prediction of the progression of cardiovascular disease in individual patients. For example, we have identified that some risk factors exhibit specific remodeling patterns, which can be computed as scores or changes in scores over time, which could guide decision on targeted interventions such as medication, lifestyle changes, or more frequent monitoring. By recognizing these patterns early, clinicians can better predict who is at higher risk of progressing to heart failure or other complications, even before clinical symptoms appear. Our novel longitudinal statistical shape atlas can capture these remodeling trajectories and could support the development of more precise, individualized treatment plans, improving patient outcomes through early detection and targeted interventions.

## 5 Limitations

Although this study used a community-based longitudinal design with a relatively large sample size and the utilization of atlas-based analysis for the evaluation of serial CMR exams, the change in CMR imaging protocol across examinations raises issues of comparability of measurements across examinations. The relatively small sample of volunteers used to train the transformation may not be representative enough to cover all pathologies. As a result, the transformation is limited to relatively normal heart shapes. Although the transformation was applied only to asymptomatic volunteers, it is unclear whether it will be as robust when used with patients who have diseases such as heart failure, where the wall becomes greatly thickened, or slow-moving blood may accentuate differences in GRE imaging. Age may also impact the transformation, but its effects are expected to be less pronounced than those of the imaging protocol. A further limitation of the study involves the necessity for a training group that underwent examination with both imaging protocols. Further work is required to develop transformations without the need for such a training set.

## 6 Conclusion

Atlas-based morphometric scores were effective in quantifying longitudinal shape changes. These shape features exhibited stronger associations with common risk factors compared to mass and volume measures. Unlike existing remodeling classifications, there is a strong and distinct regionality to the homeostatic response of the myocardium in which concentric and eccentric adaptations occur concurrently.

## Funding

This work was funded by the 10.13039/501100001505Health Research Council of New Zealand (17/234) and the engineering and Physical Sciences Research Council (EP/Z533762/1). We also acknowledge the support of NVIDIA Corporation with the donation of the Titan X Pascal GPU used for this research. MESA and the MESA SHARe project are conducted and supported by the National Heart, Lung, and Blood Institute (NHLBI) in collaboration with MESA investigators. Support for MESA is provided by contracts 75N92020D00001, HHSN268201500003I, N01-HC-95159, 75N92020D00005, N01-HC-95160, 75N92020D00002, N01-HC-95161, 75N92020D00003, N01-HC-95162, 75N92020D00006, N01-HC-95163, 75N92020D00004, N01-HC-95164, 75N92020D00007, N01-HC-95165, N01-HC-95166, N01-HC-95167, N01-HC-95168 and N01-HC-95169 from the NHLBI, and by grants UL1-TR-000040, UL1-TR-001079, and UL1-TR-001420 from the National Centre for Advancing Translational Sciences (NCATS). The authors thank the other investigators, the staff, and the participants of the MESA study for their valuable contributions. A full list of participating MESA investigators and institutions can be found at http://www.mesa-nhlbi.org.

## Author contributions

**Charlène A. Mauger:** Writing – original draft, validation, software, methodology, investigation, formal analysis, data curation, conceptualization. **Bharath Ambale-Venkatesh:** Methodology, investigation, data curation, conceptualization. **Avan Suinesiaputra:** Supervision, methodology, formal analysis, data curation, conceptualization. **David A. Bluemke:** Supervision, resources, methodology, investigation, formal analysis, data curation, conceptualization. **Colin O. Wu:** Methodology, investigation, formal analysis, data curation, conceptualization. **Joao A.C. Lima:** Writing – original draft, Methodology, Investigation, Formal analysis, Data curation, Conceptualization. **Alistair A. Young:** Writing – original draft, supervision, project administration, methodology, investigation, funding acquisition, formal analysis, data curation, conceptualization.

## Declaration of competing interests

The authors declare that they have no known competing financial interests or personal relationships that could have appeared to influence the work reported in this paper.

## Appendix A Supplementary material

.

Supplementary material Figure S1 Bias correction framework. The transformation is learnt and performed on the GRE latent space. GRE models are projected onto the GRE latent space and a correction is applied on their scores to generate SSFP-like models

.

Supplementary material Figure S2: Bias correction results for 3 different cases (columns). Top row: SSFP (wireframe) and GRE models (surface) before bias correction. Bottom row: SSFP models (wireframe) and mapped GRE models (surface) after correction

.

Supplementary material Figure S3: Parallel transport process. In the first step, the trajectory between baseline and follow-up within each participant is computed. The trajectory is then parallel transported to the common template
