# Supplementary material for: Longitudinal trajectories of left ventricular myocardial remodeling: associations with cardiovascular risk factors in the multi-ethnic study of atherosclerosis
Source: J Cardiovasc Magn Reson. 2025 Aug 22;27(2):101943. doi: 10.1016/j.jocmr.2025.101943 (PMC12745149; doi:10.1016/j.jocmr.2025.101943)
Supplement: Supplementary file 1 — Supplementary material [file mmc1.docx]

## SM1 - Correction of shape bias between protocols - Results

Table 1 reports the differences in clinical indices before and after bias correction, as well as the ground truth values. LVM was calculated at ED as the myocardial volume multiplied 1.05g/ml. The strength of agreement between SSFP shape models and debiased GRE shape models was measured using the intraclass correlation coefficient (ICC) with a two-way random effects model, single measure, and absolute agreement. A high ICC (close to 1) indicates a high similarity between the two shape models. For all measurements, higher ICC and significant decrease in bias were observed between SSFP and debiased GRE models.

The resemblance of the debiased GRE geometries to the ground truth SSFP geometries was further assessed using the point cloud structural similarity metric (PCSSM)^[[1]](#footnote-1)^*.* Coherence of the local geometry structure between the models was assessed using the Euclidean distances between a point of interest and each point belonging to its neighbourhood. For all surfaces of interest, higher structural similarity was observed, showing a higher resemblance in shape between SSFP and debiased GRE geometries after correction. The volume errors show that the leave-one-out corrected volumes and mass agree with the ground truth SSFP volumes, with an absolute bias of <3ml and <1g and respectively. Figure S2 shows examples of the bias correction on three MESA cases.

|  | | **Before correction** | | **After correction** | |
| --- | --- | --- | --- | --- | --- |
|  | | **Bias** | **ICC** | **Bias** | **ICC** |
| **End-diastolic volume (ml)** | | -8.6±9.2 | 0.91 | -2.6±9.4 | 0.94 |
| **End-systolic volume (ml)** | | -6.0±9.8 | 0.81 | -2.1±7.5 | 0.91 |
| **Mass (g)** | | 58.5±16.6 | 0.44 | 0.59±11.1 | 0.95 |
| **PCSSM** | **Endocardium at ED** | 0.86±0.04 | NA | 0.90±0.03 | NA |
|  | **Epicardium at ED** | 0.93±0.03 | NA | 0.94±0.02 | NA |
|  | **Endocardium at ES** | 080±0.05 | NA | 0.88±0.04 | NA |
|  | **Epicardium at ES** | 0.88±0.03 | NA | 0.93±0.02 | NA |

**Table 1: Average difference due to protocol bias**. ICC: intraclass correlation coefficient, GRE: gradient recalled echo, SSFP: steady-state free precession. PCSSM: Point Cloud Structural Similarity Metric.

## SM2 - Analysis of Left Ventricular Remodelling

To accurately assess how heart shape changes over time, it is essential to account for individual differences in baseline anatomy. Variability between participants often exceeds the changes observed within individuals over time. Without proper normalization, this cross-sectional variability can mask the patterns of longitudinal remodeling that are of clinical interest. To address this, all participant-specific trajectories were expressed within a shared coordinate system using a technique known as parallel transport.

Each participant's trajectory was initially defined with respect to their own baseline heart shape. To enable meaningful comparison across participants, these trajectories were mapped to a common reference. This common reference was created by computing the average of all participants' baseline heart shapes (mean baseline shape). Before averaging, the shapes were aligned using Generalized Procrustes Analysis to remove differences in position and orientation, but not in size, as heart size is an important functional parameter.

To avoid bias in the average shape, alignment was performed in two steps. First, each participant's baseline shape was aligned to a randomly selected participant. A preliminary mean shape was then calculated. All baseline shapes were then re-aligned to this preliminary mean, and a final average shape was computed. This final mean shape served as the common reference and provided a consistent anatomical reference for analyzing changes over time.

For each participant, both the baseline and follow-up heart shapes were aligned to ensure consistency in pose and orientation. Specifically, the baseline shape was aligned to the common reference, and the follow-up shape was then aligned to the transformed baseline. This hierarchical alignment approach ensured that the subsequent shape comparisons were not influenced by differences in spatial orientation or anatomical positioning.

Following alignment, each heart shape was represented in a mathematical space that accounts for shape variation. However, comparing changes between individuals in this space requires careful handling, as each trajectory resides in a slightly different geometric context. To make valid comparisons, each participant’s shape change was transferred into the shared reference frame using parallel transport.

Given the complexity of exact parallel transport for 3D shapes, we used a simplified, linear approximation. This method assumes that local shape changes can be treated as straight-line displacements from the reference shape. After calculating each participant’s trajectory, we applied this directly to the common reference. This allowed all trajectories to be expressed in a consistent and comparable manner.

To ensure the linear approximation was appropriate for our dataset, we compared distances measured using both the original curved shape space and the simplified linear transport method. The close agreement between these two measurements (y = 0.9987x + 0.00008) confirmed that the approximation was valid and that the underlying assumptions held for our application.

A visual overview of this normalization process is provided in Supplementary Figure 3. This approach enabled us to isolate and analyze individual-specific remodeling patterns, free from the confounding effects of inter-participant variability.

## SM3 - Computation of Risk-Related Remodelling Modes.

Multiple linear regression was used to compute the influence of each risk factor and confounder on LV shape trajectories. For each PCA trajectory score, a multivariate linear regression was performed as follows:

Tn ~ age + sex + race\ethnicity + ΔBSA + SBP + DBP + BMI + HDL + LDL + smoking status + Statin + Hypertension Medication + Diabetes

All variables were at follow-up, with ΔBSA being difference between baseline and follow-up. To compute the risk-related remodelling mode, the regression coefficients for all PCA trajectory scores were combined to give a weighted average of trajectory modes for each risk factor. These weighted averages defined the risk-related remodelling mode visualized below. Entries of Table 3 and 4 were calculated using the mean shapes at baseline and follow-up, computed from each risk factor individually (setting contributions from all other factors to zero).

1. E. Alexiou and E. Touradj, “Towards a point cloud structural similarity metric,” *IEEE Int. Conf. Multimed. Expo Work.*, pp. 1–6, 2020. [↑](#footnote-ref-1)
