# Supplementary figures and images for: Longitudinal trajectories of left ventricular myocardial remodeling: associations with cardiovascular risk factors in the multi-ethnic study of atherosclerosis
Source: J Cardiovasc Magn Reson. 2025 Aug 22;27(2):101943. doi: 10.1016/j.jocmr.2025.101943 (PMC12745149; doi:10.1016/j.jocmr.2025.101943)

## TRAINING

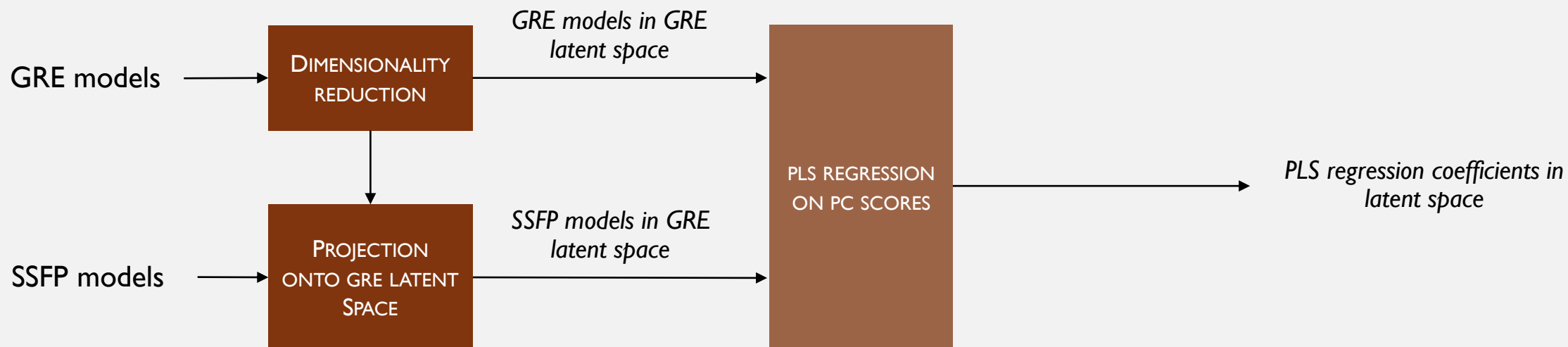

## APPLICATION

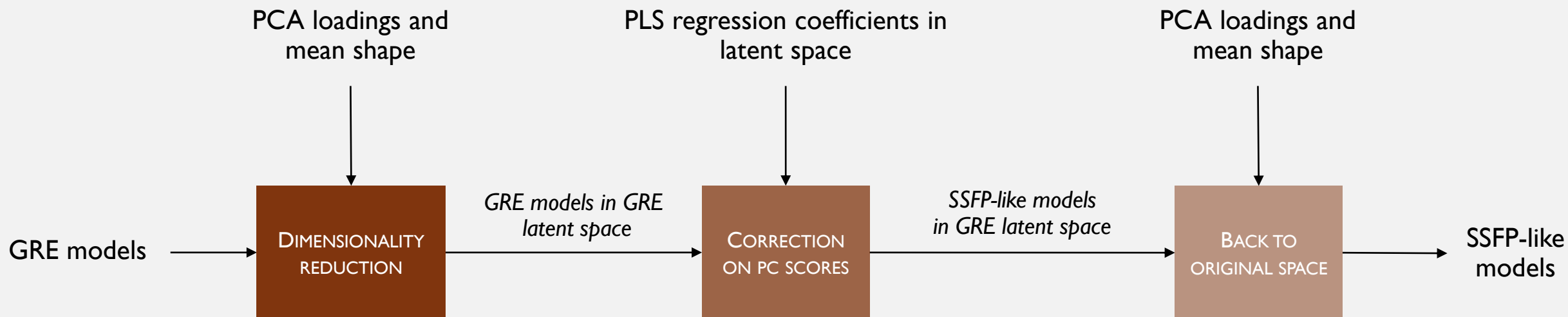

Supplement: Supplementary file 2 — Supplementary material Figure S1 Bias correction framework. The transformation is learnt and performed on the GRE latent space. GRE models are projected onto the GRE latent space and a correction is applied on their scores to generate SSFP-like models [file mmc2.pdf]

BEFORE CORRECTION

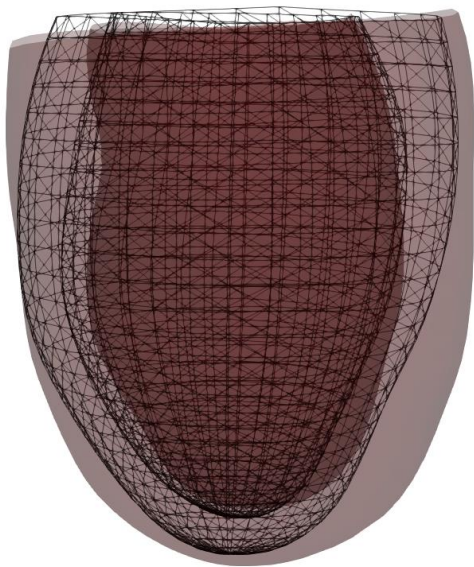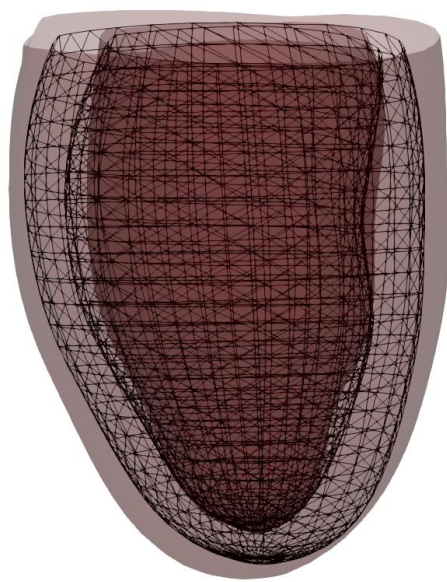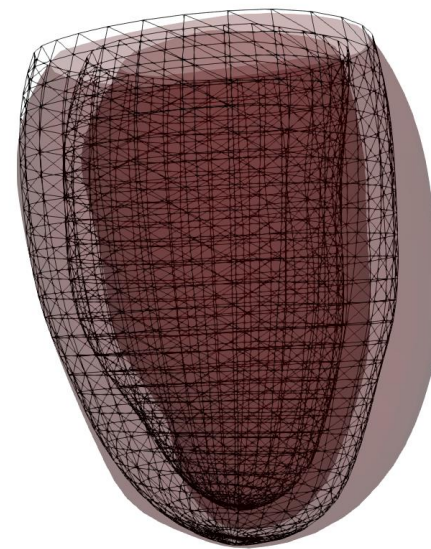

AFTER CORRECTION

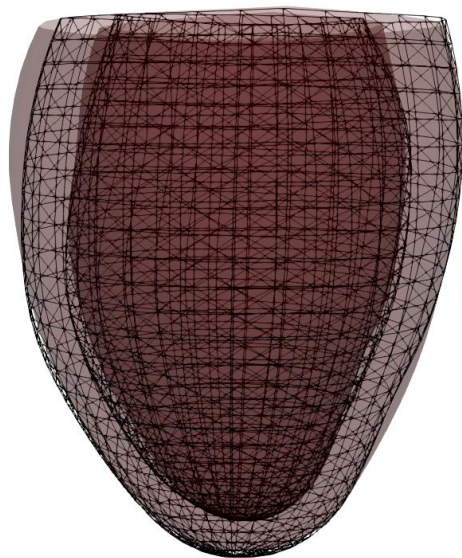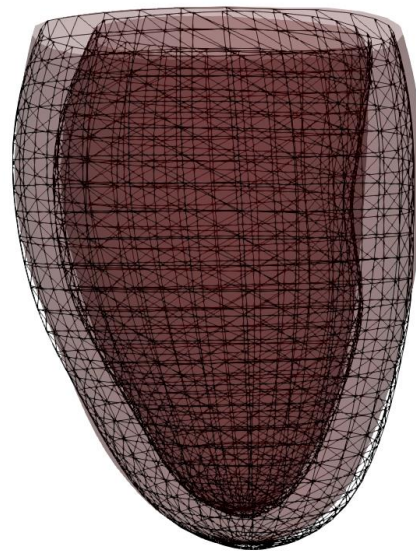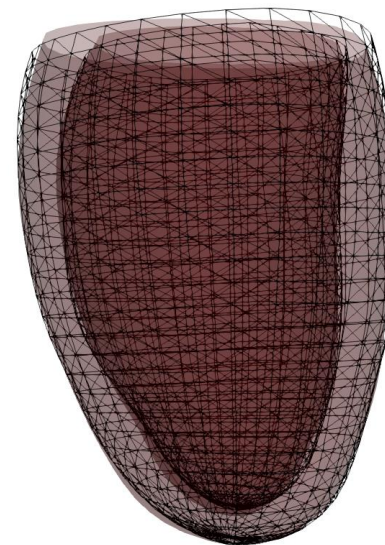

Supplement: Supplementary file 3 — Supplementary material Figure S2: Bias correction results for 3 different cases (columns). Top row: SSFP (wireframe) and GRE models (surface) before bias correction. Bottom row: SSFP models (wireframe) and mapped GRE models (surface) after correction [file mmc3.pdf]

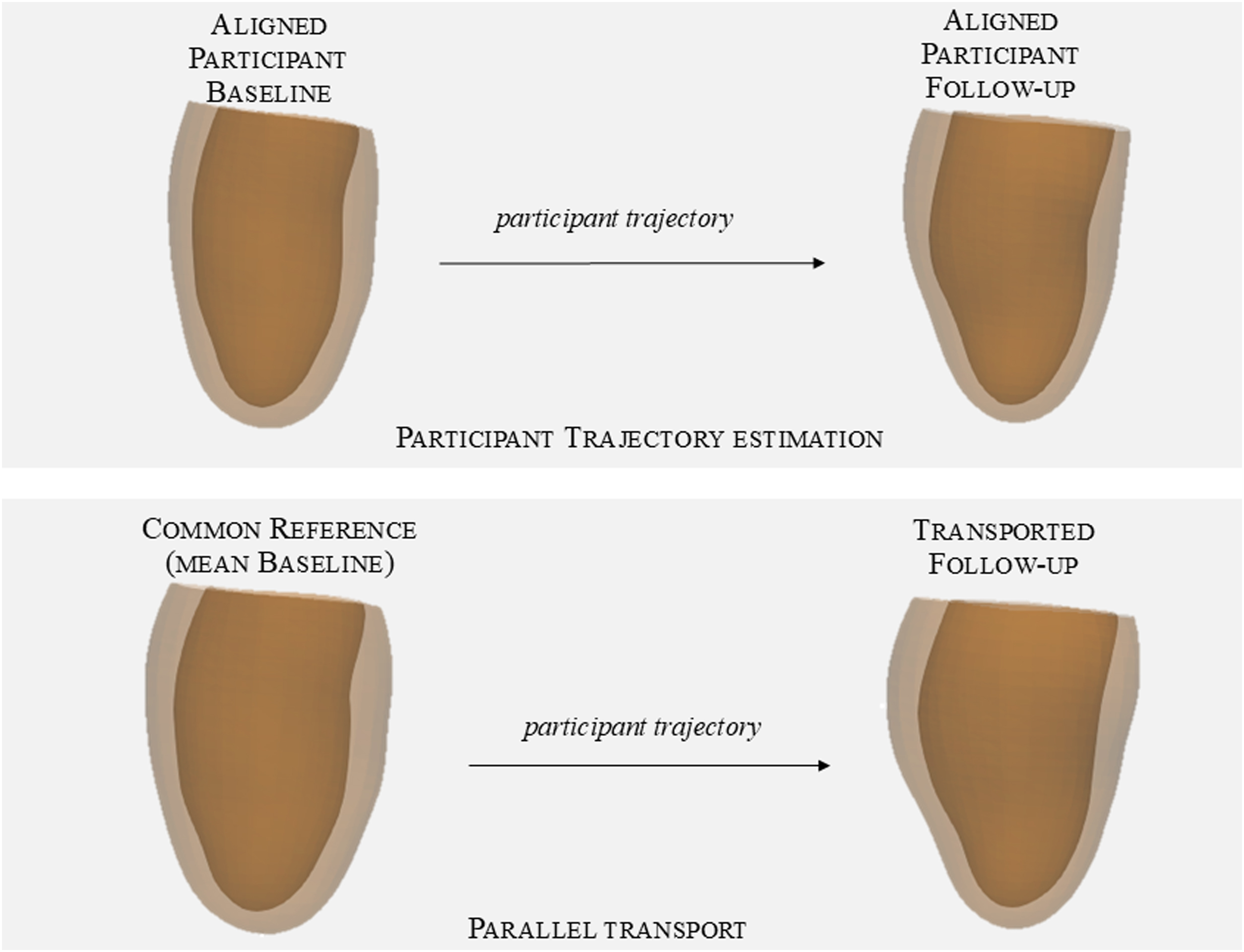

Supplement: Supplementary file 5 — Supplementary material Figure S3: Parallel transport process. In the first step, the trajectory between baseline and follow-up within each participant is computed. The trajectory is then parallel transported to the common template [file mmc5.jpg]
