# Supplementary material for: Longitudinal trajectories of left ventricular myocardial remodeling: associations with cardiovascular risk factors in the multi-ethnic study of atherosclerosis
Source: J Cardiovasc Magn Reson. 2025 Aug 22;27(2):101943. doi: 10.1016/j.jocmr.2025.101943 (PMC12745149; doi:10.1016/j.jocmr.2025.101943)
Supplement: Supplementary file 4 — Supplementary material [file mmc4.pdf]

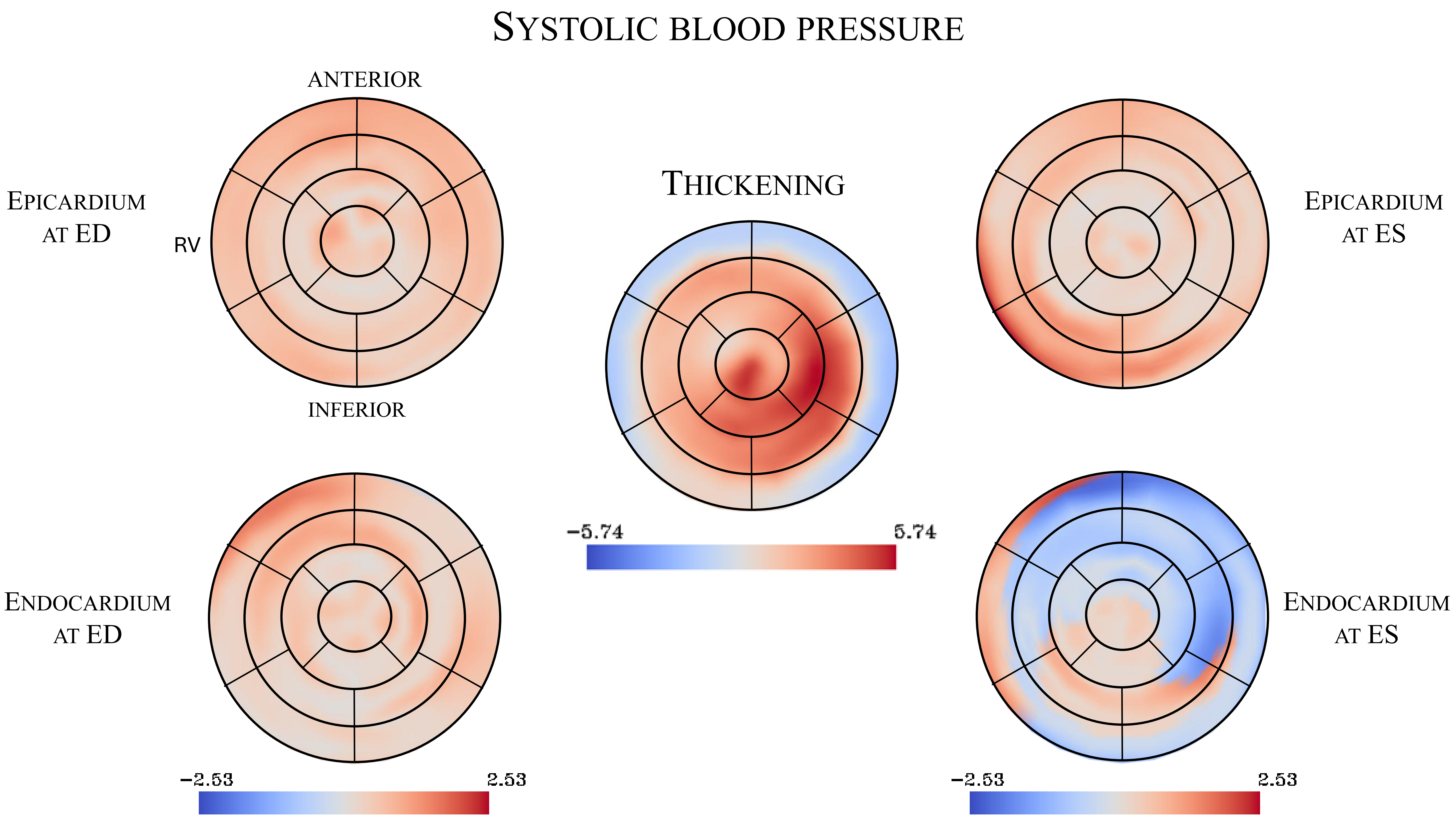
SYSTOLIC BLOOD PRESSURE
ANTERIOR
EPICARDIUM AT ED
RV
INFERIOR
THICKENING
EPICARDIUM AT ES
−5.74
5.74
ENDOCARDIUM AT ED
ENDOCARDIUM AT ES
−2.53
2.53
−2.53
2.53

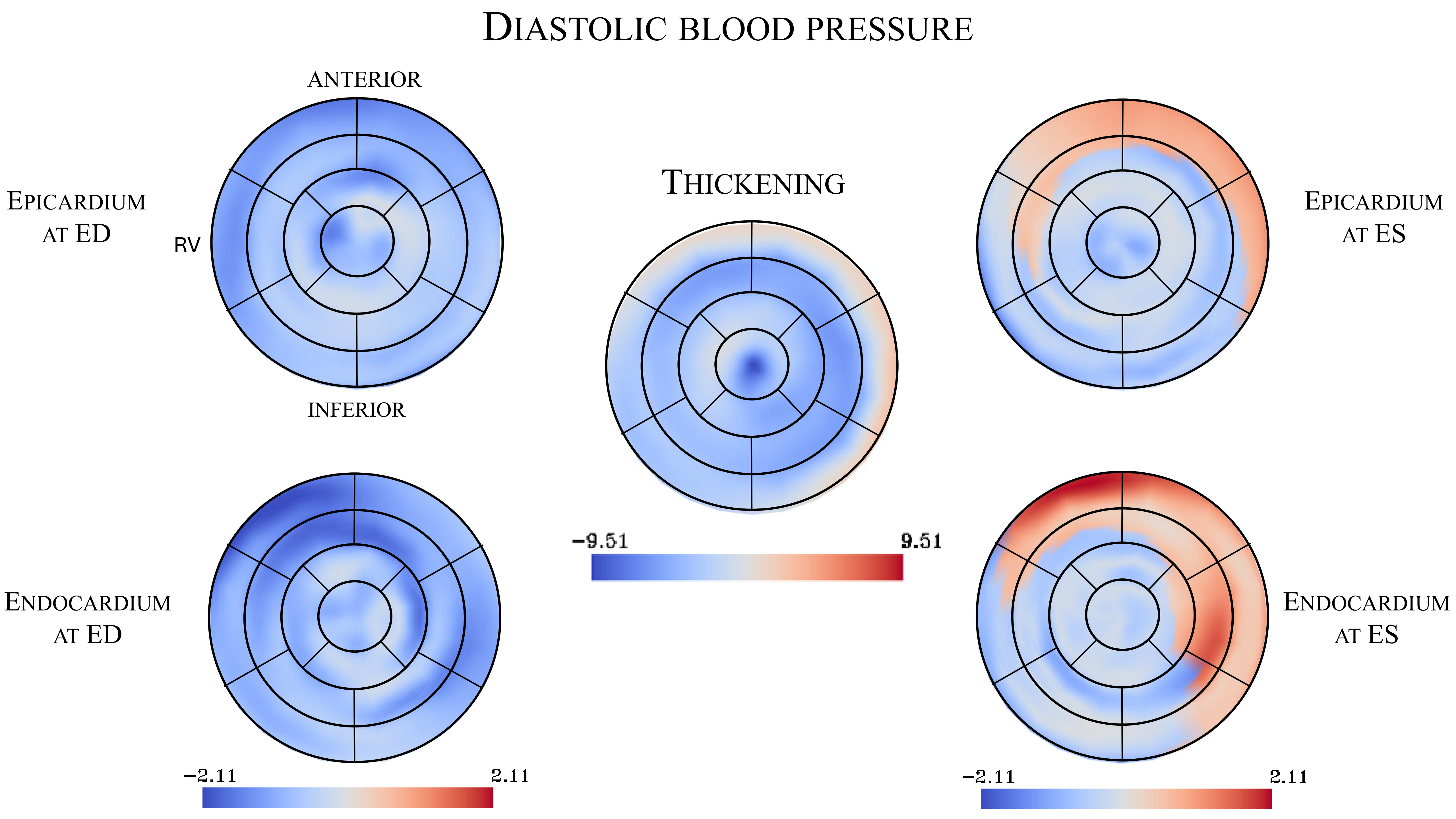
DIASTOLIC BLOOD PRESSURE
ANTERIOR
EPICARDIUM AT ED
RV
INFERIOR
THICKENING
EPICARDIUM AT ES
−9.51
9.51
ENDOCARDIUM AT ED
ENDOCARDIUM AT ES
−2.11
2.11
−2.11
2.11

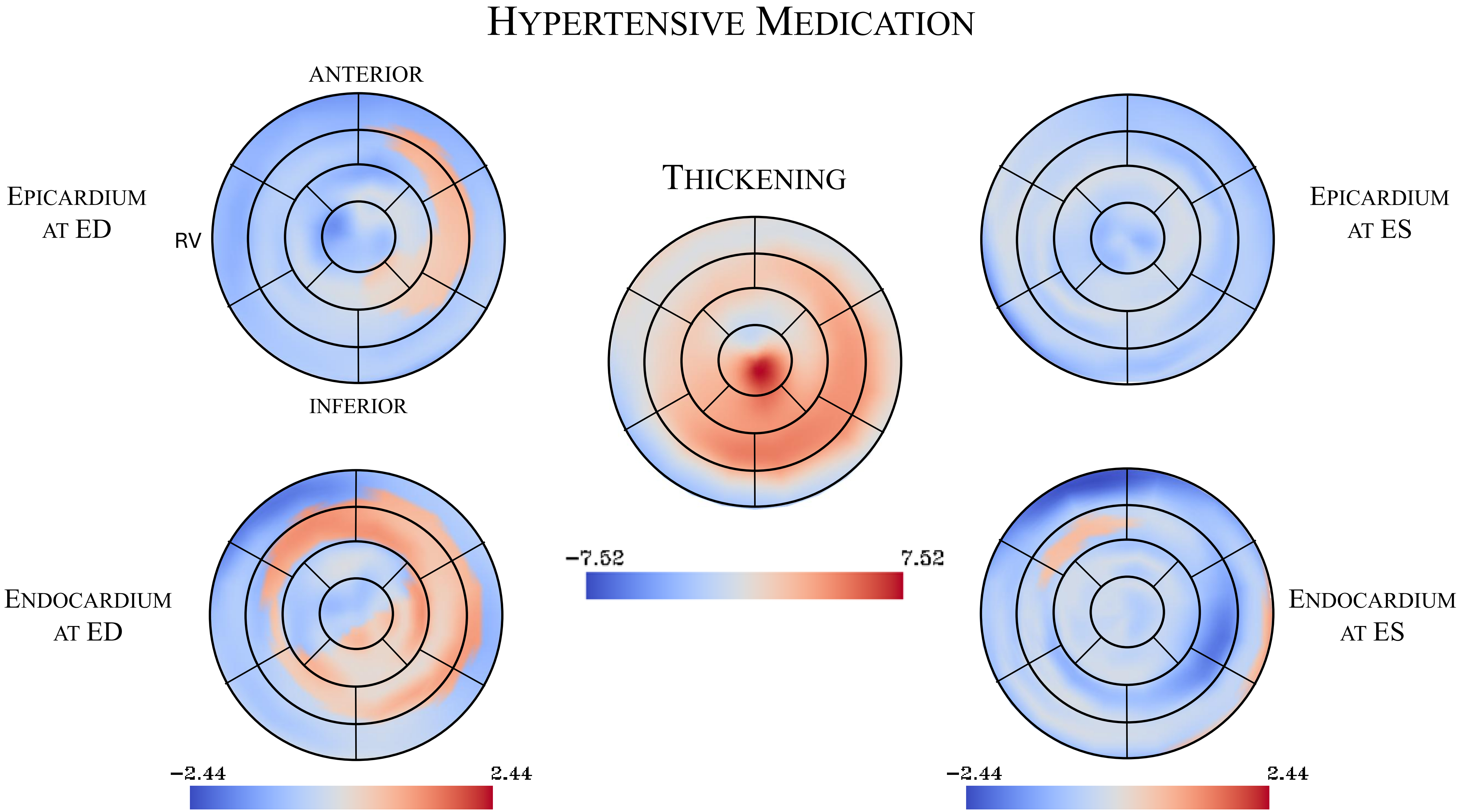

HYPERTENSIVE MEDICATION
ANTERIOR
INFERIOR
RV
EPICARDIUM AT ED
THICKENING
EPICARDIUM AT ES
ENDOCARDIUM AT ED
ENDOCARDIUM AT ES
−7.52
7.52
−2.44
2.44
−2.44
2.44

High-Density Lipoproteins

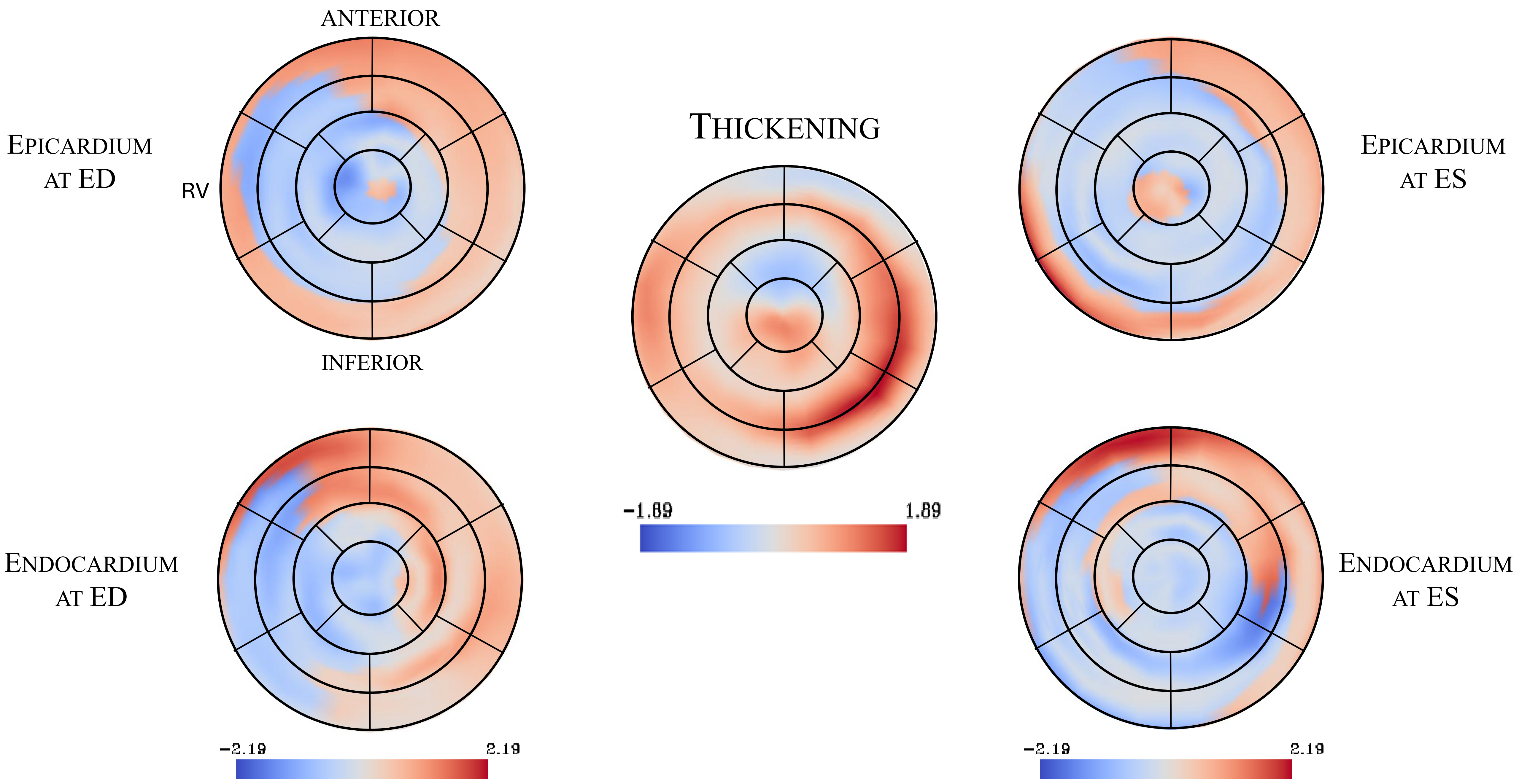

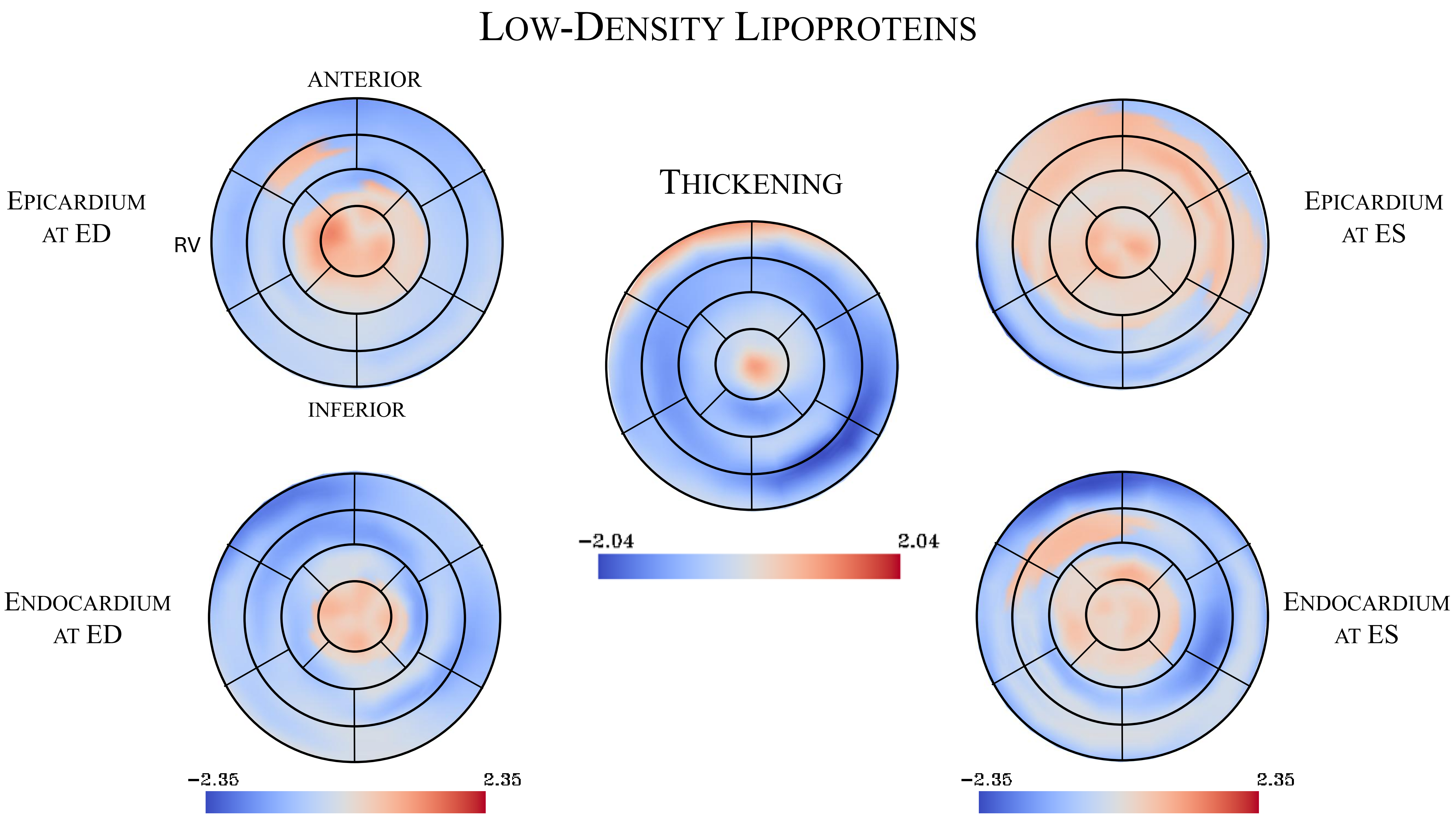
LOW-DENSITY LIPOPROTEINS
ANTERIOR
EPICARDIUM AT ED
RV
INFERIOR
THICKENING
EPICARDIUM AT ES
−2.04
2.04
ENDOCARDIUM AT ED
ENDOCARDIUM AT ES
−2.35
2.35
−2.35
2.35

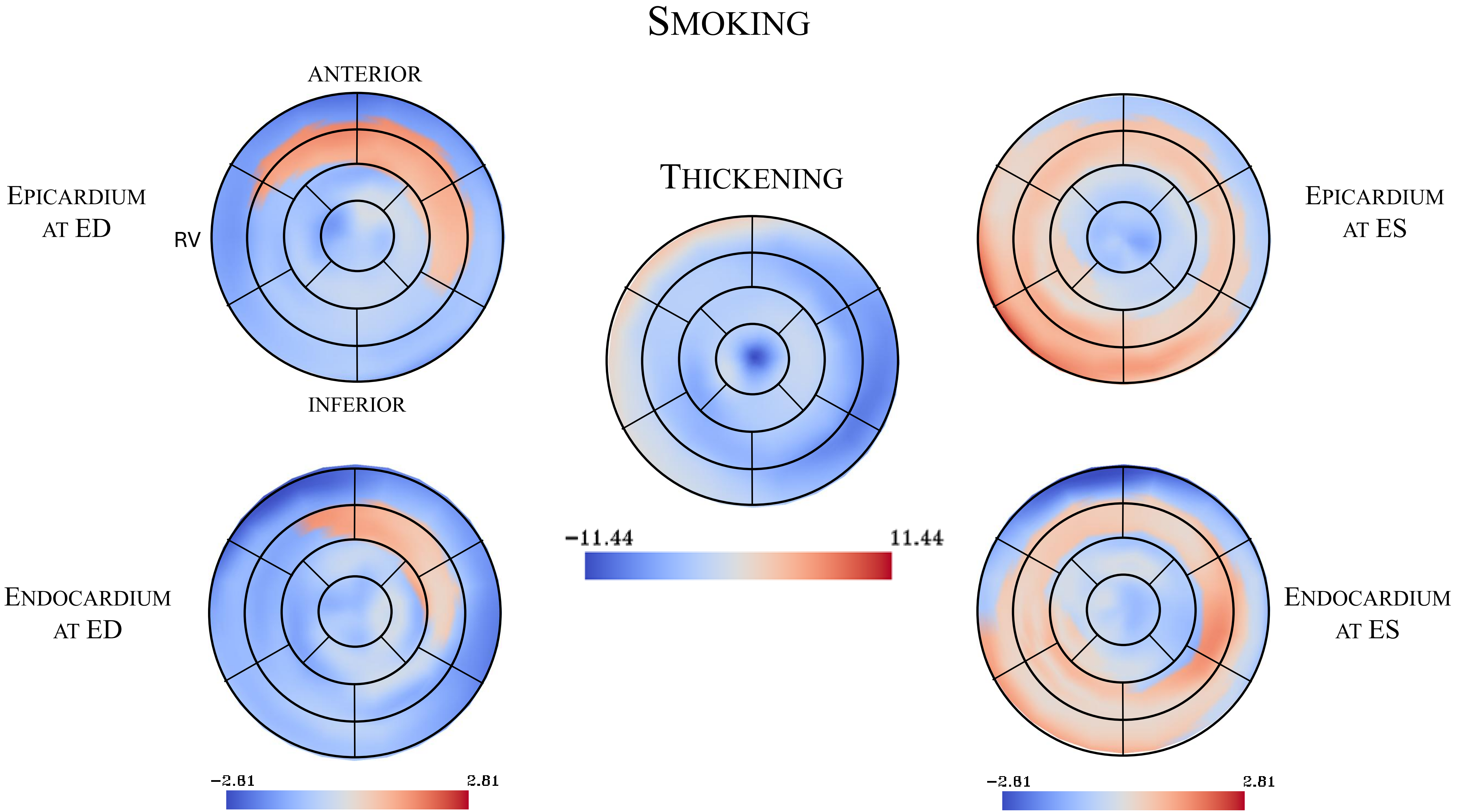
SMOKING
ANTERIOR
EPICARDIUM AT ED
RV
INFERIOR
THICKENING
EPICARDIUM AT ES
−11.44
11.44
ENDOCARDIUM AT ED
ENDOCARDIUM AT ES
−2.81
2.81
−2.81
2.81

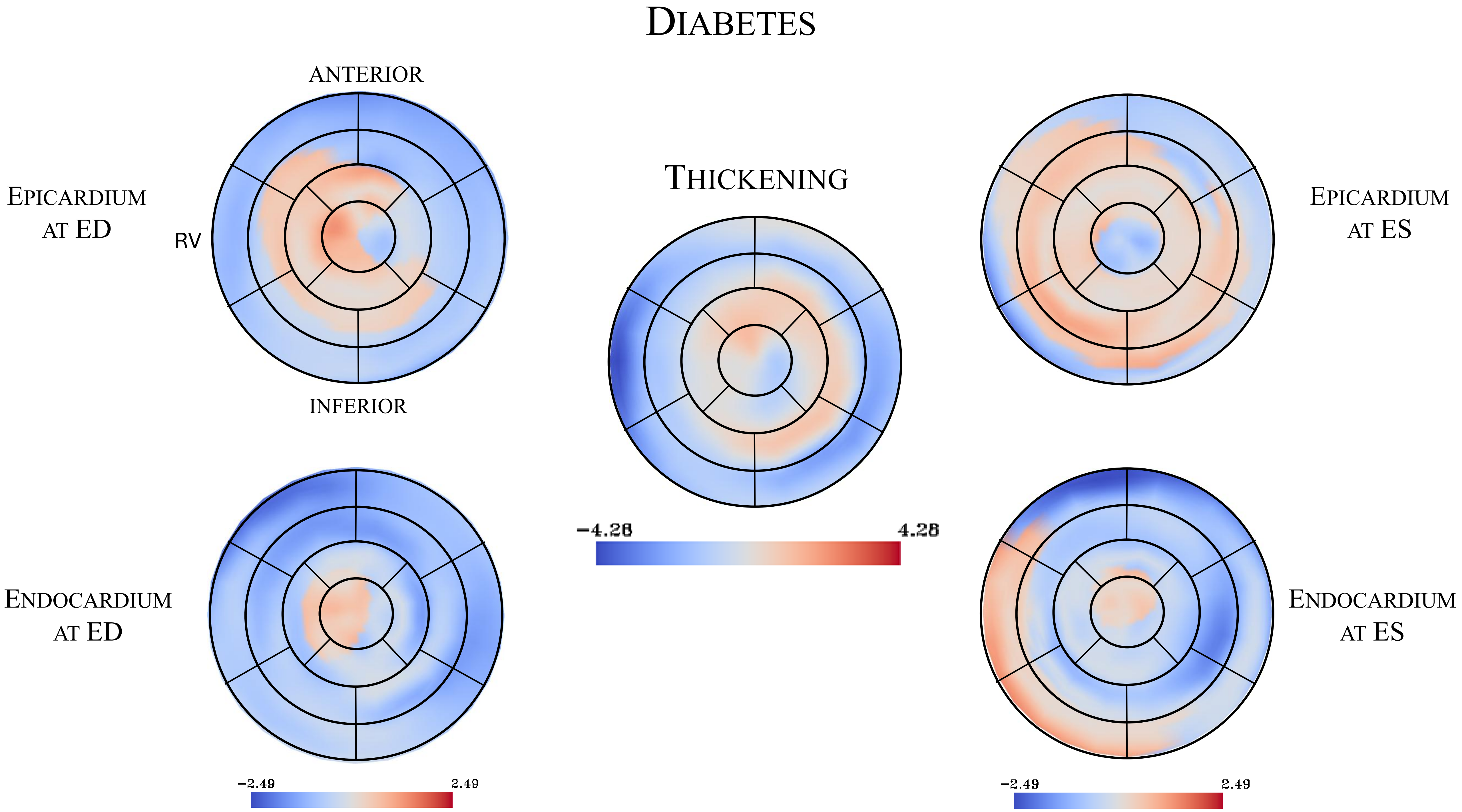
DIABETES
ANTERIOR
EPICARDIUM AT ED
RV
INFERIOR
THICKENING
EPICARDIUM AT ES
−4.28
4.28
ENDOCARDIUM AT ED
ENDOCARDIUM AT ES
−2.49
2.49
−2.49
2.49

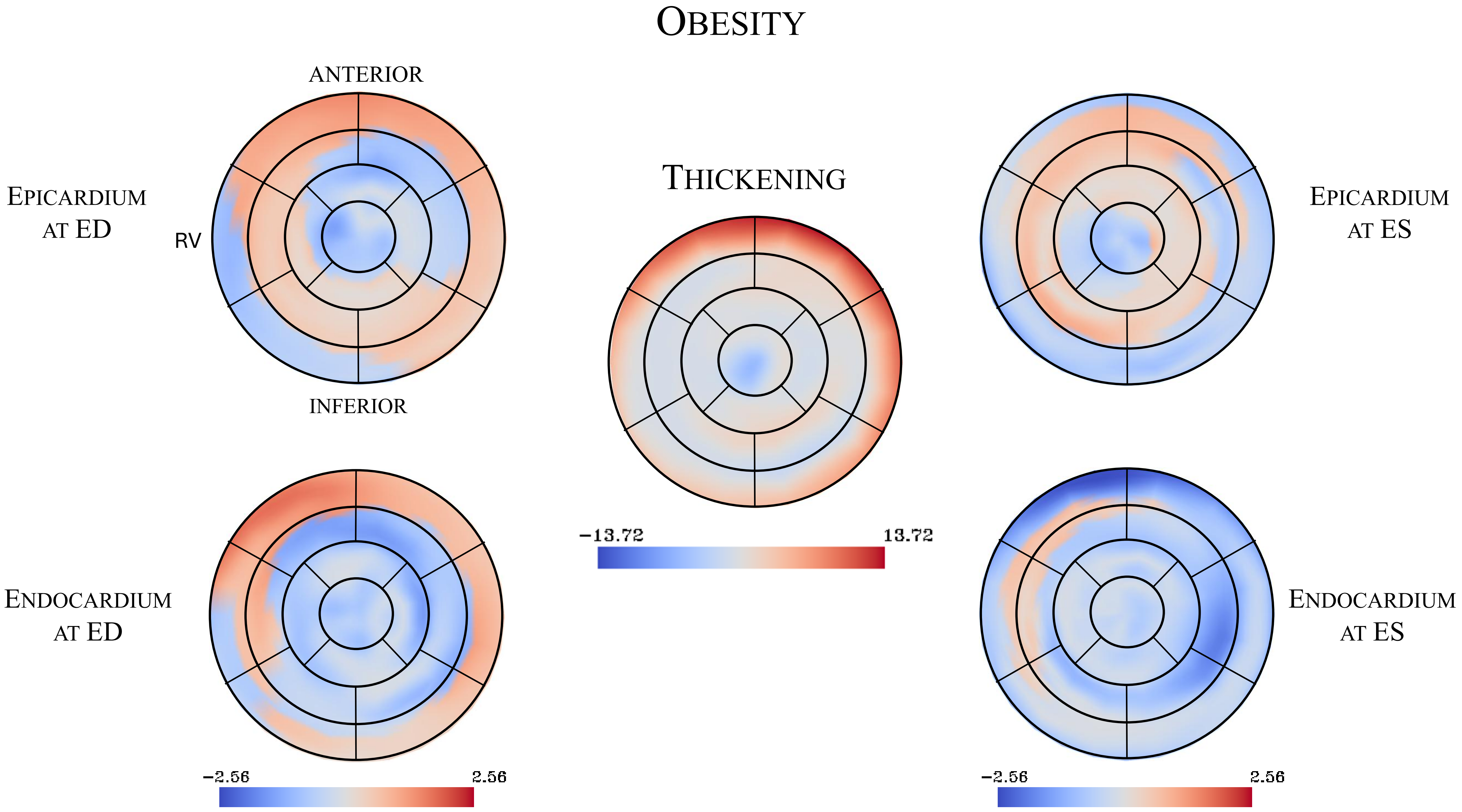

OBESITY
ANTERIOR
EPICARDIUM AT ED
RV
INFERIOR
THICKENING
EPICARDIUM AT ES
−13.72
13.72
ENDOCARDIUM AT ED
ENDOCARDIUM AT ES
−2.56
2.56
−2.56
2.56

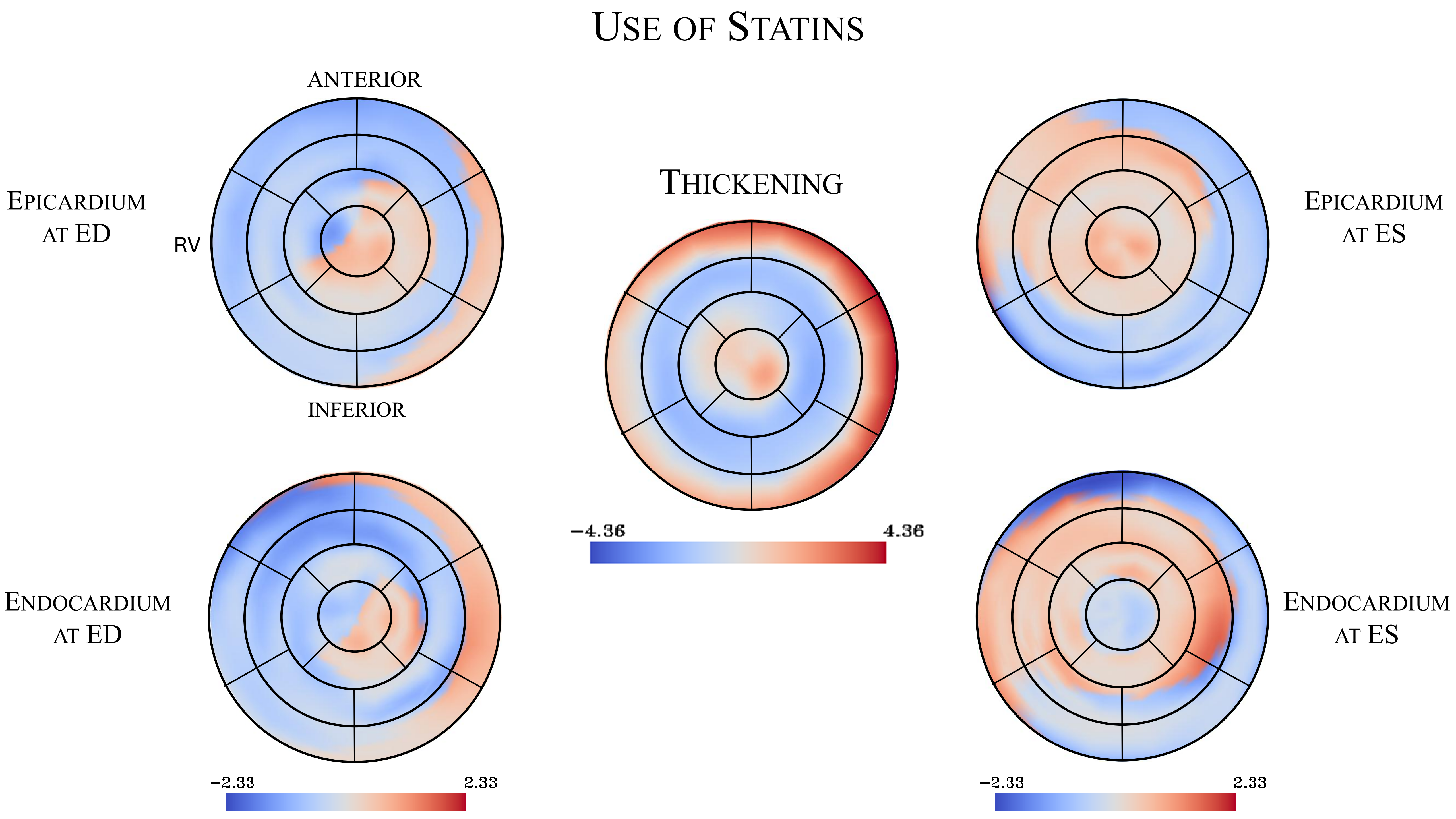
USE OF STATINS
ANTERIOR
INFERIOR
RV
EPICARDIUM AT ED
ENDOCARDIUM AT ED
THICKENING
−4.36
4.36
EPICARDIUM AT ES
ENDOCARDIUM AT ES
−2.33
2.33
−2.33
2.33
